# Pim1 Serves as a Therapeutic Target for Inflammatory Arthritis via Mitochondrial Metabolism and Th17 Cell Differentiation

**DOI:** 10.34133/research.1137

**Published:** 2026-02-27

**Authors:** Zepeng Su, Yipeng Zeng, Qibo Li, Jinteng Li, Guan Zheng, Weihao Zhang, Zipeng Xiao, Zibin Chen, Yangfeng Lin, Ziqian Liu, Yanfeng Wu, Jiajie Lin, Wenhui Yu, Zhongyu Xie

**Affiliations:** ^1^ Department of Orthopedics, The Eighth Affiliated Hospital of Sun Yat-Sen University, Shenzhen 518000, China.; ^2^ Center for Biotherapy, The Eighth Affiliated Hospital of Sun Yat-Sen University, Shenzhen 518000, China.; ^3^ Guangdong Provincial Clinical Research Center for Orthopedic Diseases, The Eighth Affiliated Hospital of Sun Yat-Sen University, Shenzhen 518000, China.

## Abstract

Inflammatory arthritis, mainly including rheumatoid arthritis (RA) and ankylosing spondylitis (AS), is a group of chronic progressive autoimmune diseases that destroy joints. T helper 17 (Th17) cells are extensively involved in the joint inflammation as well as bone and cartilage destruction of these diseases. Previously, proviral integration site for Moloney-murine leukemia virus 1 (Pim1) was reported to be involved in various autoimmune diseases by mediating the proinflammatory effects of T cells. However, the pathological effect and the therapeutic potential of Pim1 in inflammatory arthritis remain elusive. The present study demonstrated that Pim1 expression was elevated in CD4^+^ T cells locally and systemically in patients with RA or AS and in 2 mice models of inflammatory arthritis. Conditional knockdown of Pim1 in CD4^+^ T cells (Pim1 cKO) or using the Pim1 inhibitor AZD1208 alleviated the development of inflammatory arthritis in association with decreasing the proportion of Th17 cells. In vitro experiments involving inhibition and overexpression confirmed the promoting effect of Pim1 on Th17 cell differentiation. Mechanistically, Pim1 phosphorylated mitochondrial calcium uptake protein 1 to increase mitochondrial calcium influx, which subsequently activated mitochondrial oxidative phosphorylation and promoted Th17 cell differentiation. Through molecular docking and dynamic simulation, nilotinib, a Food-and-Drug-Administration-approved drug, was identified as a selective substitute for the currently clinically nonapproved Pim1 inhibitors, which impeded Th17 cell differentiation and was well tolerated during the treatment of Pim1 cKO mice and 2 inflammatory arthritis mouse models. Our study contributes to a better understanding of the mechanism through which Pim1 promotes Th17 cell differentiation and advances the clinical application of Pim1 as an effective target for treating inflammatory arthritis.

## Introduction

Inflammatory arthritis, mainly including rheumatoid arthritis (RA) and ankylosing spondylitis (AS), is a group of chronic progressive inflammatory diseases characterized by the destruction of bones and joints [1]. Despite some differences in clinical manifestations, aberrant differentiation of T helper 17 (Th17) cells is involved in accelerating the pathological process of both RA and AS [2,3]. Th17 cells, through the secretion of interleukin-17A (IL-17A), IL-17F, IL-26, and granulocyte-macrophage colony-stimulating factor (GM-CSF), recruit macrophages and neutrophils to the inflamed joint tissue and mediate cartilage destruction and bone erosion, which are the pathological characteristics of inflammatory arthritis [3–5]. Therefore, elucidating the mechanism of the aberrant Th17 cell differentiation in inflammatory arthritis will contribute to a better understanding of the pathogenesis and lead to improved treatments for this type of arthritis.

The proviral integration site for Moloney-murine leukemia virus 1 (Pim1) is a serine/threonine phosphokinase [6]. Pim1 has received considerable attention because of its antiapoptotic and cell-cycle-promoting effects, which accelerate the development of T cell tumors [7]. Further research revealed that Pim1 is found to be involved in the pathogenesis of autoimmune diseases by mediating cytokine-dependent signaling in T cells, which is important for T cell differentiation [8,9]. A prior study has showed that Pim kinase exerts therapeutic effects on early RA by regulating the differentiation potential of T cells [10]. However, both the underlying mechanism by which Pim1 affects Th17 cell proportion and its involvement in inflammatory arthritis remain to be elucidated.

Mitochondrial metabolism, especially oxidative phosphorylation (OXPHOS), participates in regulating the differentiation and function of multiple immune cells, including Th17 cells, and therefore contributes to the pathogenesis of inflammatory diseases [11]. OXPHOS not only provides energy for the vital activities that support Th17 cell differentiation and the generation of pathogenic cytokines but also enhances the mitochondrial metabolic adaptability and increases the resistance of Th17 cells to apoptosis, which supports their continued pathogenic effects [12,13]. Although Pim1, which regulates the functions of immune cells through mitochondrial reactive oxygen species (ROS) and fatty acid metabolism, has not been reported to regulate OXPHOS, it participates in maintaining the mitochondrial calcium (mito-Ca^2+^) homeostasis, which is an essential regulator of OXPHOS activity [14–17]. Whether Pim1 modulates Th17 cell differentiation through mitochondrial OXPHOS warrants further investigation.

The present study revealed the elevated Pim1 expression in CD4^+^ T cells from patients with inflammatory arthritis and its mouse models. Specifically intercepting Pim1 alleviated the development of inflammatory arthritis by impeding Th17 cell differentiation. Mechanistically, Pim1 inhibited Th17 cell differentiation by phosphorylating mito-Ca^2+^ uptake 1 (MICU1), increasing mito-Ca^2+^ influx and subsequently activating mitochondrial OXPHOS. In addition, the Food and Drug Administration (FDA)-approved drug nilotinib was identified through molecular docking and dynamic simulation, which bound to Pim1 to suppress its activity and demonstrated therapeutic effects on inflammatory arthritis. Our study contributes to a better understanding of the mechanism through which Pim1 promotes Th17 cell differentiation and accelerates the pathological process of inflammatory arthritis and supports the clinical application of Pim1 as an effective target for the treatment of inflammatory arthritis.

## Results

### Pim1 expression is increased in CD4^+^ T cells in inflammatory arthritis

**Fig. 1.**
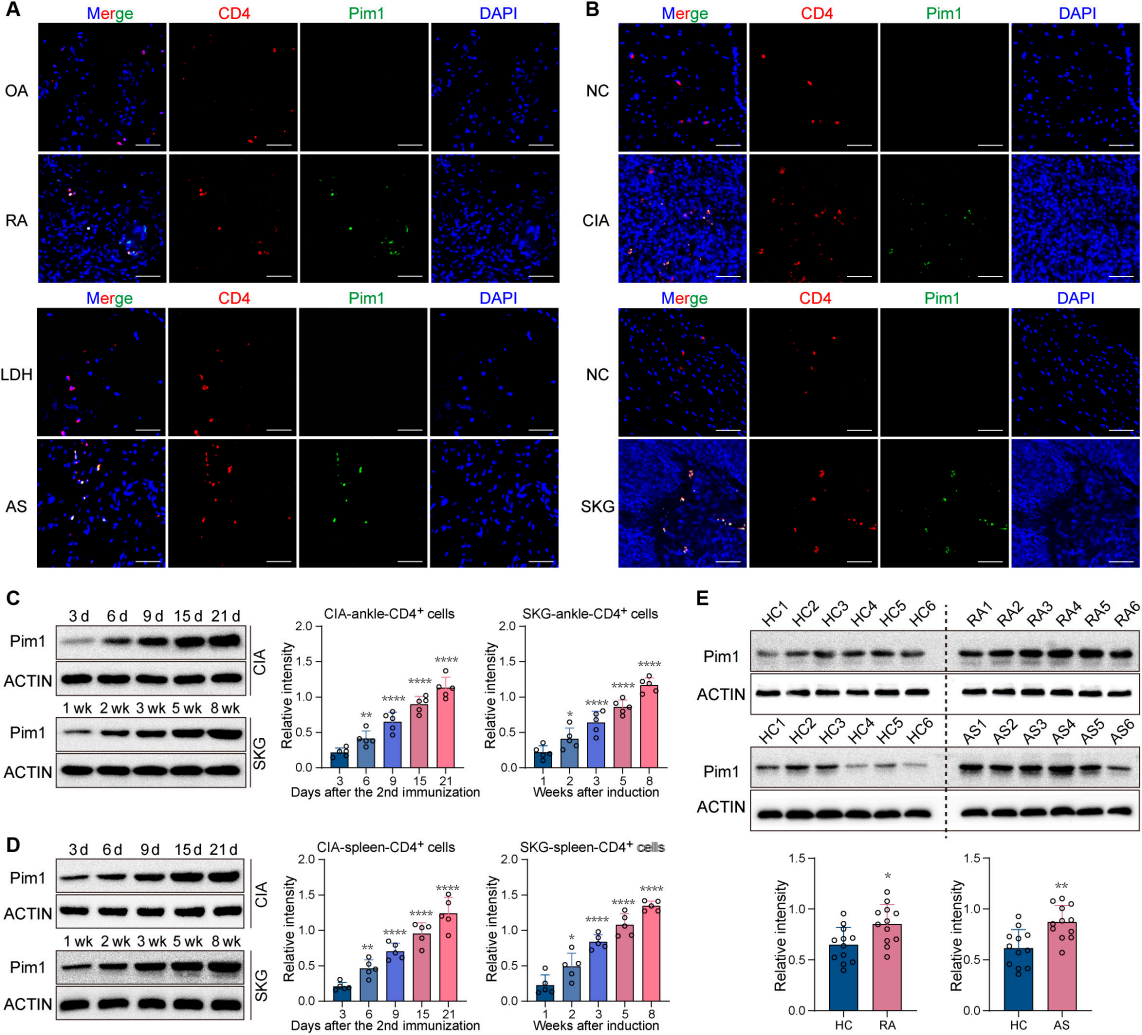
Pim1 expression is increased in CD4^+^ T cells in inflammatory arthritis. (A) Representative immunofluorescence (IF) images showing Pim1 expression in CD4^+^ cells in the OA and RA synovium and the LDH and AS entheses. Scale bars, 50 μm. DAPI, 4′,6-diamidino-2-phenylindole. (B) Representative immunofluorescence images showing Pim1 expression in CD4^+^ cells in ankle tissues from CIA and SKG arthritis model mice and the corresponding normal controls. Scale bars, 50 μm. (C and D) Relative protein levels of Pim1 in CD4^+^ T cells in ankle tissues (C) and spleens (D) of CIA and SKG arthritis mice over time following arthritis induction (*n* = 5). (E) Relative protein levels of Pim1 in peripheral blood CD4^+^ T cells from patients with RA and AS and HCs (*n* = 12). The data were statistically analyzed via one-way analysis of variance (ANOVA), followed by Bonferroni’s post hoc comparisons test (C and D) and 2-tailed Student’s *t* test (E).

Pim1 expression was measured in CD4^+^ T cells and CD68^+^ macrophages, 2 major types of pathogenic cells involved in inflammatory arthritis. Compared with that in patients with osteoarthritis (OA) and those with lumbar disc herniation (LDH), Pim1 expression was increased in infiltrative CD4^+^ T cells in the synovium from RA patients and the ligament entheses from patients with AS (Fig. 1A). However, Pim1 expression in CD68^+^ macrophages in the RA synovium and AS entheses was similar to that in the controls (Fig. S1A). Pim1 expression was also increased in infiltrating CD4^+^ T cells in inflammatory ankle tissues from collagen-induced arthritis (CIA) and SKG mice, 2 animal models of inflammatory arthritis, compared with those from the undiseased mice (Fig. 1B). In inflamed ankle tissues from CIA and SKG mice, Pim1 expression in F4/80^+^ macrophages was comparable to that in normal controls (Fig. S1B). With increase in time after arthritis induction, Pim1 expression was progressively up-regulated in CD4^+^ T cells in the ankle tissues of CIA and SKG mice, whereas Pim1 expression in F4/80 macrophages did not change markedly (Fig. 1C and Fig. S1C). Pim1 expression was also increased progressively in the splenic CD4^+^ T cells of the CIA and SKG mice over time after arthritis induction (Fig. 1D). In addition, Pim1 expression was elevated in the peripheral blood CD4^+^ T cells of patients with RA and AS compared with healthy controls (HCs) (Fig. 1E). These findings suggest that Pim1 expression in CD4^+^ T cells is both locally and systemically increased in inflammatory arthritis.

### Specifically intercepting Pim1 alleviates the development of inflammatory arthritis by impeding Th17 cell differentiation

**Fig. 2.**
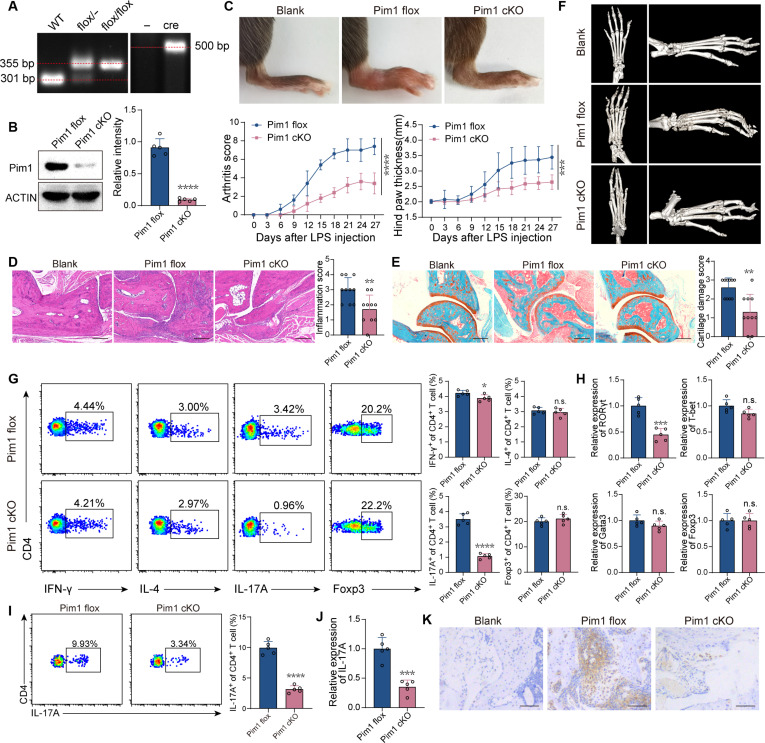
Specifically intercepting Pim1 alleviates the development of inflammatory arthritis by impeding Th17 cell differentiation. (A) Identification of Pim1 flox and CD4-cre gene. WT, wild type. (B) Relative protein levels of Pim1 in CD4^+^ T cells isolated from Pim1 flox and Pim1 cKO mice. (C) Macroscopic images of the ankles, arthritis scores, and hind paw thicknesses of Pim1 flox and Pim1 cKO CIA mice (*n* = 5). (D and E) Representative histological images with H&E staining (D) and safranin O-fast green staining (E) of ankles from Pim1 flox and Pim1 cKO CIA mice (*n* = 10). Scale bars, 200 μm. (F) Representative micro-CT images of the ankles of Pim1 flox and Pim1 cKO CIA mice. (G) Frequencies of IFN-γ^+^, IL-4^+^, IL-17A^+^, and Foxp3^+^ cells among CD4^+^ cells in the spleens of Pim1 flox and Pim1 cKO CIA mice (*n* = 5). (H) Relative mRNA expression of RORγt, T-bet, GATA3, and Foxp3 in the ankles of Pim1 flox or Pim1 cKO CIA mice (*n* = 5). (I) Frequencies of IL-17A^+^ cells among CD4^+^ cells in the ankles of Pim1 flox and Pim1 cKO CIA mice (*n* = 5). (J) Relative mRNA expression of IL-17A in the ankles of Pim1 flox or Pim1 cKO CIA mice (*n* = 5). (K) Representative immunohistochemical (IHC) images showing IL-17A expression in the ankle tissues of Pim1 flox and Pim1 cKO CIA mice. Scale bars, 50 μm. The data were statistically analyzed via 2-tailed Student’s *t* test (B, D, E, and G to J) and 2-way repeated-measures ANOVA (C).

To explore the role of Pim1 in inflammatory arthritis, we established mice with conditional knockout of Pim1 in CD4^+^ cells (Pim1 cKO) along with their control (Pim1 flox) and subjected them to arthritis induction. The genetic identification of Pim1 flox and Pim1 cKO mice was shown as Fig. 2A. Pim1 expression was barely detectable in the CD4^+^ T cells of Pim1 cKO mice (Fig. 2B). Swelling and redness were less severe in the joints of Pim1 cKO mice than in those of Pim1 flox mice, as indicated by the arthritis score and hind paw thickness (Fig. 2C). Consistently, Pim1 flox mice developed severe inflammatory infiltration, cartilage erosion, and bone destruction in the joints after arthritis induction, which was significantly inhibited in Pim1 cKO mice (Fig. 2D to F). Although the ratio of Th1 cells was slightly decreased and that of Th2 cells or regulatory T cells (Treg cells) was unchanged, the Th17 cell proportion in splenocytes was significantly decreased in Pim1 cKO mice (Fig. 2G). Consistent results were obtained when measuring the expression of the key transcription factors for the CD4^+^ T cell subpopulations in the ankle tissues (Fig. 2H). In addition, the ratio of Th17 cells in ankle tissues was decreased in Pim1 cKO mice compared with that in Pim1 flox mice (Fig. 2I). The salient expression of IL-17A in joint tissues of Pim1 flox mice following arthritis induction was decreased in Pim1 cKO mice, as measured by real-time quantitative polymerase chain reaction (RT-qPCR) and immunohistochemical staining (Fig. 2J and K). To confirm the results above, we orally administered AZD1208, a Pim1 inhibitor, in CIA and SKG mice before arthritis induction to determine its preventive effects (PRE group) and after arthritis induction to determine its therapeutic effects (TRE group). In the CIA and SKG mice, joint swelling was less severe, and arthritis scores were lower in the PRE and TRE groups than in the negative control (NC) group (Fig. S2A and B). In addition, CIA mice exhibited smaller hind paw thickness, and SKG mice exhibited a slower onset of arthritis in the PRE and TRE groups than in the NC group (Fig. S2A and B). Consistently, inflammatory infiltration, cartilage erosion, and bone destruction were also improved in PRE and TRE groups (Fig. S2C to E). The frequency of Th17 cells among splenic CD4^+^ T cells was decreased in the PRE and TRE groups compared with the NC group (Fig. S2F). Moreover, the ratio of Th17 cells in CD4^+^ T cells and the IL-17A expression in the joint tissues were both decreased in PRE and TRE groups (Fig. S2G and H). These results demonstrated that specifically inhibiting Pim1 showed preventive and therapeutic effects on inflammatory arthritis, in association with the impairment of Th17 cell differentiation.

### Pim1 promotes Th17 cell differentiation of CD4^+^ T cells in vitro

**Fig. 3.**
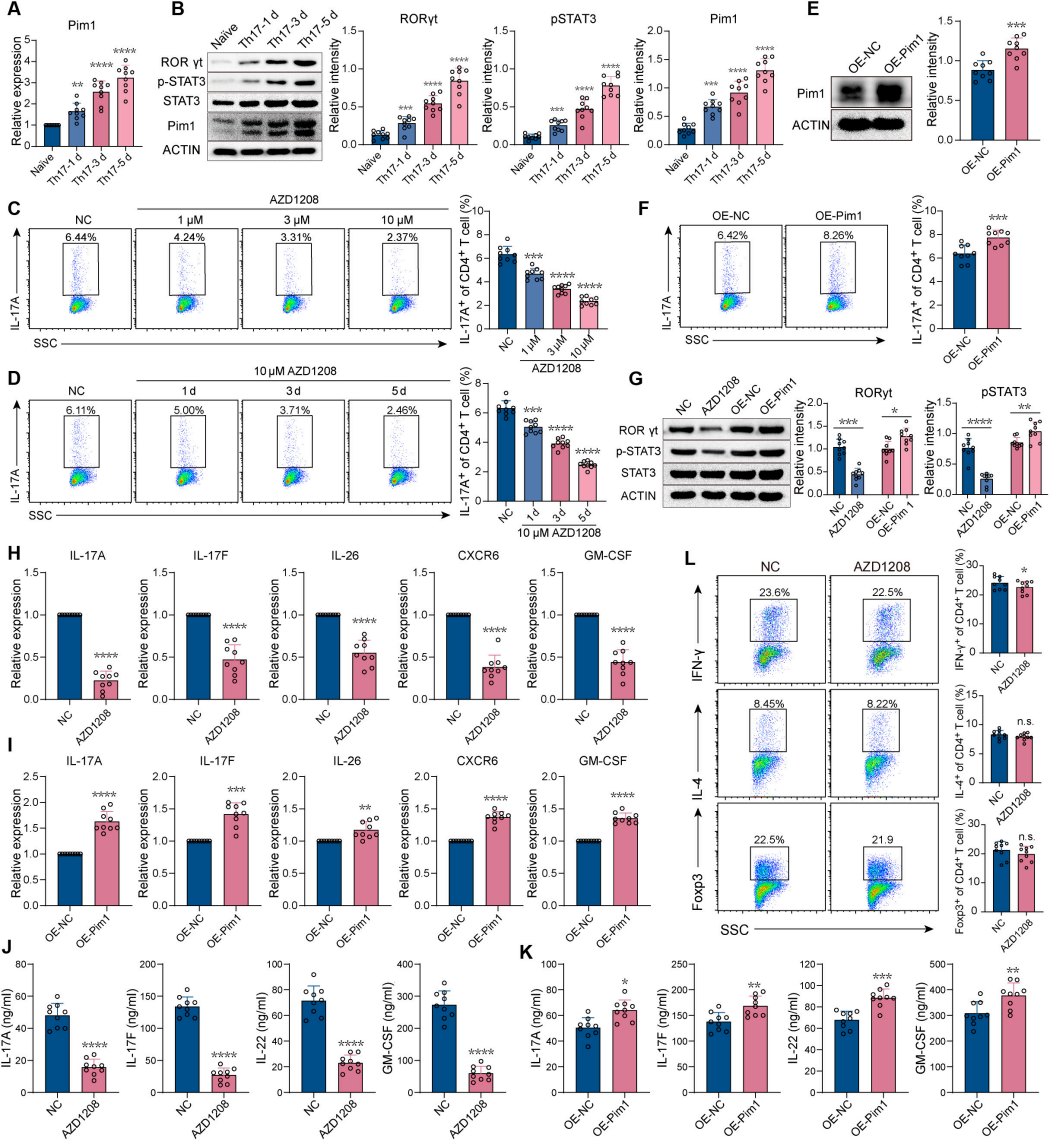
Pim1 promotes Th17 cell differentiation in vitro. (A) Relative mRNA levels of Pim1 during Th17 cell differentiation (*n* = 9). (B) Relative protein levels of pSTAT3, RORγt, and Pim1 during Th17 cell differentiation (*n* = 9). (C and D) Frequency of Th17 cells among CD4^+^ cells after AZD1208 treatment (*n* = 9). (E) Relative protein levels of Pim1 among CD4^+^ T cells overexpressing (OE) vector or Pim1 (*n* = 9). (F) Frequency of Th17 cells in CD4^+^ cells after overexpressing vector or Pim1 (*n* = 9). (G) Relative protein levels of RORγt and pSTAT3 after AZD1208 treatment or Pim1 overexpression (*n* = 9). (H and I) Relative mRNA levels of Th17-cell-associated pathogenic genes after AZD1208 treatment (H) or Pim1 overexpression (I) (*n* = 9). (J and K) Concentration of IL-17A, IL-17F, IL-22, and GM-CSF in the cell supernatant after AZD1208 treatment (J) or Pim1 overexpression (K). (L) Frequencies of Th1, Th2, and Treg cells among CD4^+^ T cells after treatment with AZD1208 (*n* = 9). The data were statistically analyzed via one-way ANOVA, followed by Bonferroni’s post hoc comparisons (A to D) and paired *t* test (E to L).

The Pim1 expression at the mRNA and protein levels was progressively up-regulated concomitantly with Th17 cell differentiation, which was consistent with the increased expression of retinoid-related orphan receptor-gamma (RORγt) and phosphorylated signal transducers and activators of transcription 3 (pSTAT3) (Fig. 3A and B). Inhibiting Pim1 with AZD1208 reduced the frequency of Th17 cells (Fig. 3C and D), whereas Pim1 overexpression increased the Th17 cell proportion in CD4^+^ T cells (Fig. 3E and F). The protein levels of RORγt and pSTAT3 were also inhibited by AZD1208 and increased by Pim1 overexpression, respectively (Fig. 3G). Inhibiting Pim1 suppressed the expression of a series of Th17-cell-associated pathogenic genes, including IL-17A, IL-17F, IL-26, CXCR6, and GM-CSF, while overexpressing Pim1 promoted the expression of these cytokines (Fig. 3H and I). Similarly, Pim1 inhibition suppressed and Pim1 overexpression promoted the secretion of IL-17A, IL-17F, IL-22, and GM-CSF (Fig. 3J and K). However, AZD1208 slightly reduced the frequency of Th1 cells, with no significant effect on the frequency of Th2 or Treg cells (Fig. 3L). These results showed that Pim1 predominantly promotes the differentiation of Th17 cells in CD4^+^ T cells.

### Pim1 promotes Th17 cell differentiation by enhancing OXPHOS activity

**Fig. 4.**
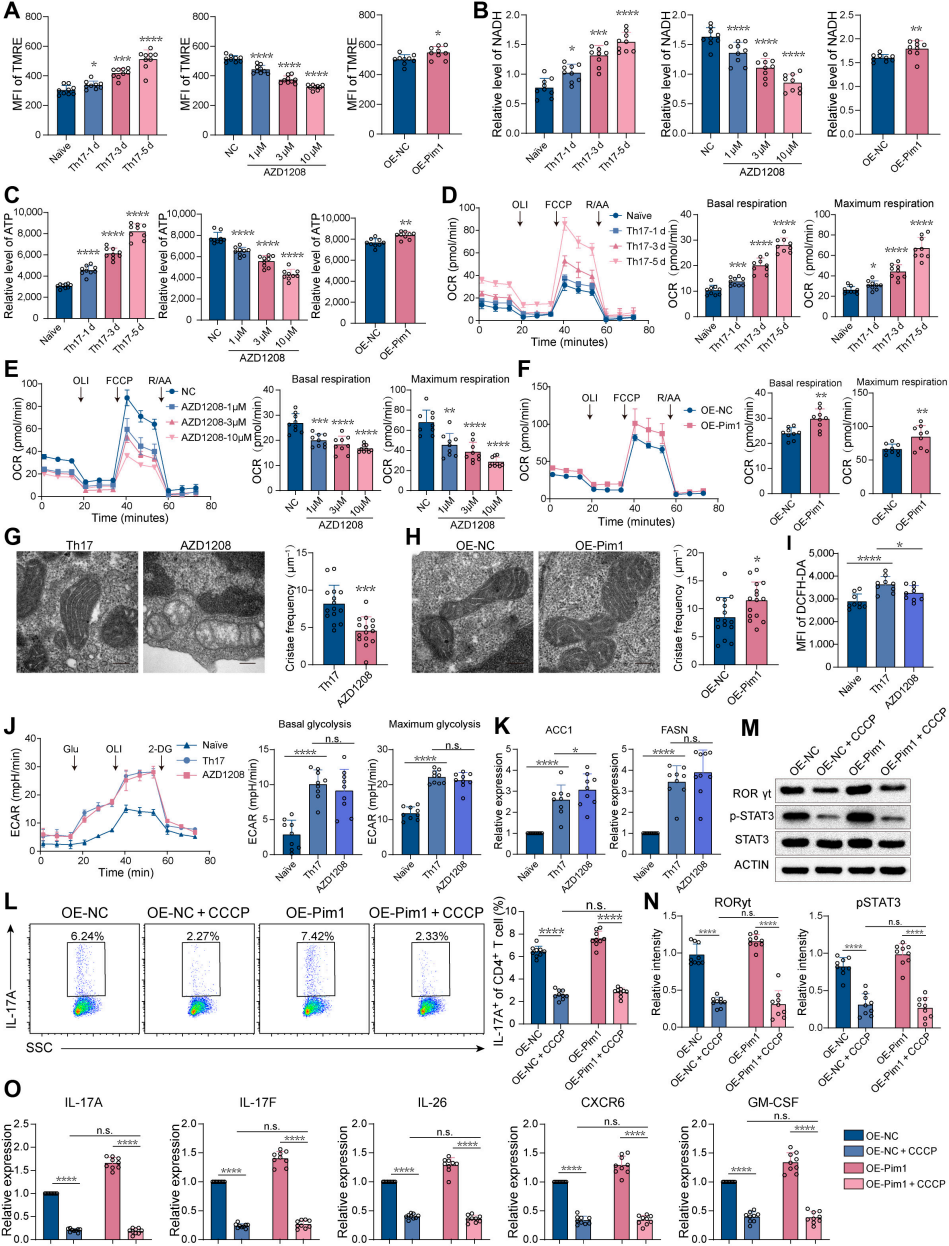
Pim1 regulates Th17 cell differentiation through OXPHOS. (A to C) The MFI of TMRE (A) and the relative levels of NADH (B) and ATP (C) in cells at different stages of Th17 cell differentiation and in cells treated with different concentrations of AZD1208 and overexpressing Pim1 (*n* = 9). (D to F) OCR of cells at different stages of Th17 cell differentiation (D), cells treated with different concentrations of AZD1208 (E), and cells overexpressing Pim1 (F) (*n* = 9). OLI, oligomycin. (G and H) Mitochondrial cristae frequency in cells treated with AZD1208 (G) and cells overexpressing Pim1 (H) (*n*=15). Scale bars, 200 nm. (I) ROS levels of cells in naïve, Th17, and AZD1208 groups (*n* = 9). (J) ECAR of cells in the naïve, Th17, and AZD1208 groups (*n* = 9). (K) Relative mRNA levels of the key enzymes involved in fatty acid synthesis of cells in naïve, Th17, and AZD1208 groups (*n* = 9). (L) Frequency of Th17 cells among CD4^+^ cells after CCCP treatment in the presence or absence of Pim1 overexpression (*n* = 9). (M and N) Relative protein levels of RORγt and pSTAT3 in cells treated with CCCP in the presence or absence of Pim1 overexpression (*n* = 9). (O) Relative mRNA levels of Th17-cell-associated pathogenic genes in cells treated with CCCP in the presence or absence of Pim1 overexpression (*n* = 9). The data were statistically analyzed via one-way ANOVA, followed by Bonferroni’s post hoc comparisons (A to E and I to O), paired *t* test (A to C and F), and 2-tailed Student’s *t* test (G and H)

To determine the mechanism through which Pim1 promotes Th17 cell differentiation, we conducted RNA sequencing using naïve CD4^+^ T cells (naïve group), cells cultured under Th17 cell polarization conditions (Th17 group), and then treated with AZD1208 (AZD1208 group). Principal components analysis and cluster heatmaps revealed that the gene expression profiles markedly differed among the 3 groups (Fig. S3A and B). The volcano plot showed that the expression of 5,617 genes was altered with 3,111 genes up-regulated and 2,506 genes down-regulated during Th17 cell differentiation. The expression of 634 genes was altered, with 114 up-regulated and 520 down-regulated by Pim1 inhibition under Th17 cell differentiation conditions (Fig. S3C). A total of 324 genes were identified for their opposite tendencies between the Th17 versus naïve comparisons and the AZD1208 versus Th17 comparisons (Fig. S3D). Gene Ontology Biological Process analysis revealed that the 324 genes were enriched in Th17 cell differentiation and several inflammatory-disease-related pathways including RA (Fig. S3E). The expression of the representative cytokine and their receptor genes, including IL-17A and IL-17F, increased after Th17 cell differentiation condition but was inhibited by AZD1208 treatment (Fig. S3F). Furthermore, the OXPHOS pathways were enriched in different databases according to gene set enrichment analysis of the Th17 versus naïve group and the AZD1208 versus Th17 group (Fig. S3G). During Th17 cell differentiation, the OXPHOS activity, as determined via the mitochondrial membrane potential and the levels of reduced nicotinamide adenine dinucleotide (NADH) and adenosine triphosphate (ATP), gradually increased, which was reduced by AZD1208 treatment and enhanced by Pim1 overexpression (Fig. 4A to C). Similar trends were observed in the basal and maximal oxygen consumption rates (OCRs; Fig. 4D to F). In addition, upon AZD1208 treatment, mitochondrial cristae frequency was decreased, which was increased by Pim1 overexpression (Fig. 4G and H). The levels of ROS also increased in Th17 cells as compared with naïve CD4^+^ T cells, which were decreased by AZD1208 (Fig. 4I). However, AZD1208 did not significantly inhibit effect on glycolysis or fatty acid synthesis (Fig. 4J and K). Furthermore, the OXPHOS inhibitor CCCP significantly decreased the proportion of Th17 cells, even under Pim1 overexpression conditions (Fig. 4L). The expression of RORγt, pSTAT3, and Th17-cell-associated pathogenic genes, showed consistent trends (Fig. 4M to O). These results suggest that Pim1 promotes Th17 cell differentiation by enhancing OXPHOS activity.

### Pim1 facilitates mito-Ca^2+^ influx to activate OXPHOS through phosphorylating MICU1

**Fig. 5.**
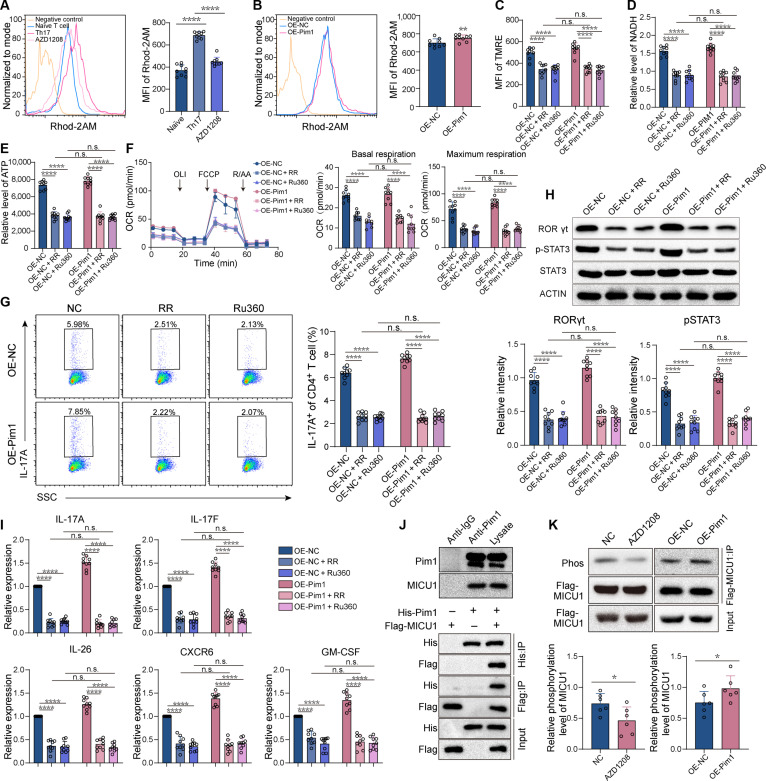
Pim1 promotes OXPHOS by facilitating mito-Ca^2+^ influx. (A) MFI of Rhod-2AM of cells in the naïve, Th17, and AZD1208 groups (*n* = 9). (B) MFI of Rhod-2AM of cells overexpressing the vector or Pim1 (*n* = 9). (C to E) The MFI of TMRE (C) and the relative levels of NADH (D) and ATP (E) in cells treated with RR or Ru360 in the presence or absence of Pim1 overexpression (*n* = 9). (F) OCR of cells treated with RR or Ru360 in the presence or absence of Pim1 overexpression (*n* = 9). (G) Frequency of Th17 cells among CD4^+^ cells after treatment with RR or Ru360 in the presence or absence of Pim1 overexpression (*n* = 9). (H) Relative protein levels of RORγt and pSTAT3 in cells treated with RR or Ru360 in the presence or absence of Pim1 overexpression (*n* = 9). (I) Relative mRNA levels of Th17-cell-associated pathogenic genes in cells treated with RR or Ru360 in the presence or absence of Pim1 overexpression (*n* = 9). (J) IP showing the interaction of Pim1 and MICU1 in Th17 cells (top) and HEK-293T cells (bottom). (K) Phosphorylation level of MICU1 in cells after AZD1208 treatment or Pim1 overexpression (*n* = 6). The data were statistically analyzed via one-way ANOVA, followed by Bonferroni’s post hoc comparisons (A and C to I) and paired *t* test (B and K).

OXPHOS activity is supported by mito-Ca^2+^ [17]. During Th17 cell differentiation, the concentration of mito-Ca^2+^ increased, which was decreased by AZD1208 treatment but increased by Pim1 overexpression (Fig. 5A and B). The mito-Ca^2+^ concentration is regulated by mito-Ca^2+^ influx, and ruthenium red (RR) and Ru360 inhibited mito-Ca^2+^ influx and then decreased the mito-Ca^2+^ concentration [18]. Upon RR or Ru360 treatment, the mitochondrial potential, NADH and ATP levels, along with the basal and maximum OCR were all decreased. Similar inhibitory effects were observed under Pim1 overexpression condition (Fig. 5C to F). Furthermore, RR and Ru360 reduced the frequency of Th17 cells in CD4^+^ T cells to the same extent regardless of Pim1 overexpression, together with the reduced expression of RORγt, pSTAT3, and Th17-cell-associated pathogenic genes (Fig. 5G to I). Pim1 is a phosphokinase that elevates protein phosphorylation levels and then regulates the activity of mito-Ca^2+^ influx-related proteins [19]. MICU1, a protein that promotes mito-Ca^2+^ influx [20], was identified to interact with Pim1 through immunoprecipitation (IP), followed by protein mass spectrometry (Fig. 5J). In addition, the phosphorylation level of MICU1 was decreased by AZD1208 and increased by Pim1 overexpression (Fig. 5K). To explore whether Pim1 mediates mito-Ca^2+^ influx through MICU1, MICU1 was knockdown in CD4^+^ T cells. The knockdown efficiency of MICU1 in CD4^+^ T cells was verified (Fig. S4A). Knockdown of MICU1 inhibited mito-Ca^2+^ influx to an equal level in the presence and absence of Pim1 overexpression (Fig. S4B). Moreover, MICU1 knockdown decreased the mean fluorescence intensity (MFI) of tetramethylrhodamine ethyl ester (TMRE), the levels of NADH and ATP, and the OCR regardless of Pim1 overexpression (Fig. S4C to F). Importantly, MICU1 knockdown significantly inhibited Th17 cell differentiation, as indicated by flow cytometry analysis of the proportion of IL-17A^+^ cells in CD4^+^ T cells, Western blot analysis of the expression of RORγt and pSTAT3, and RT-qPCR analysis of the expression of IL-17A, IL-17F, IL-22, CXCR6, and GM-CSF. In addition, in the presence of Pim1 overexpression, MICU1 knockdown inhibited Th17 cell differentiation to an extent comparable to that observed in the absence of Pim1 overexpression (Fig. S4G to I). These results suggest that Pim1 enhances mito-Ca^2+^ influx and activates OXPHOS through MICU1, potentially by increasing the phosphorylation level of MICU1.

### Molecular docking and dynamic simulations of Pim1

**Fig. 6.**
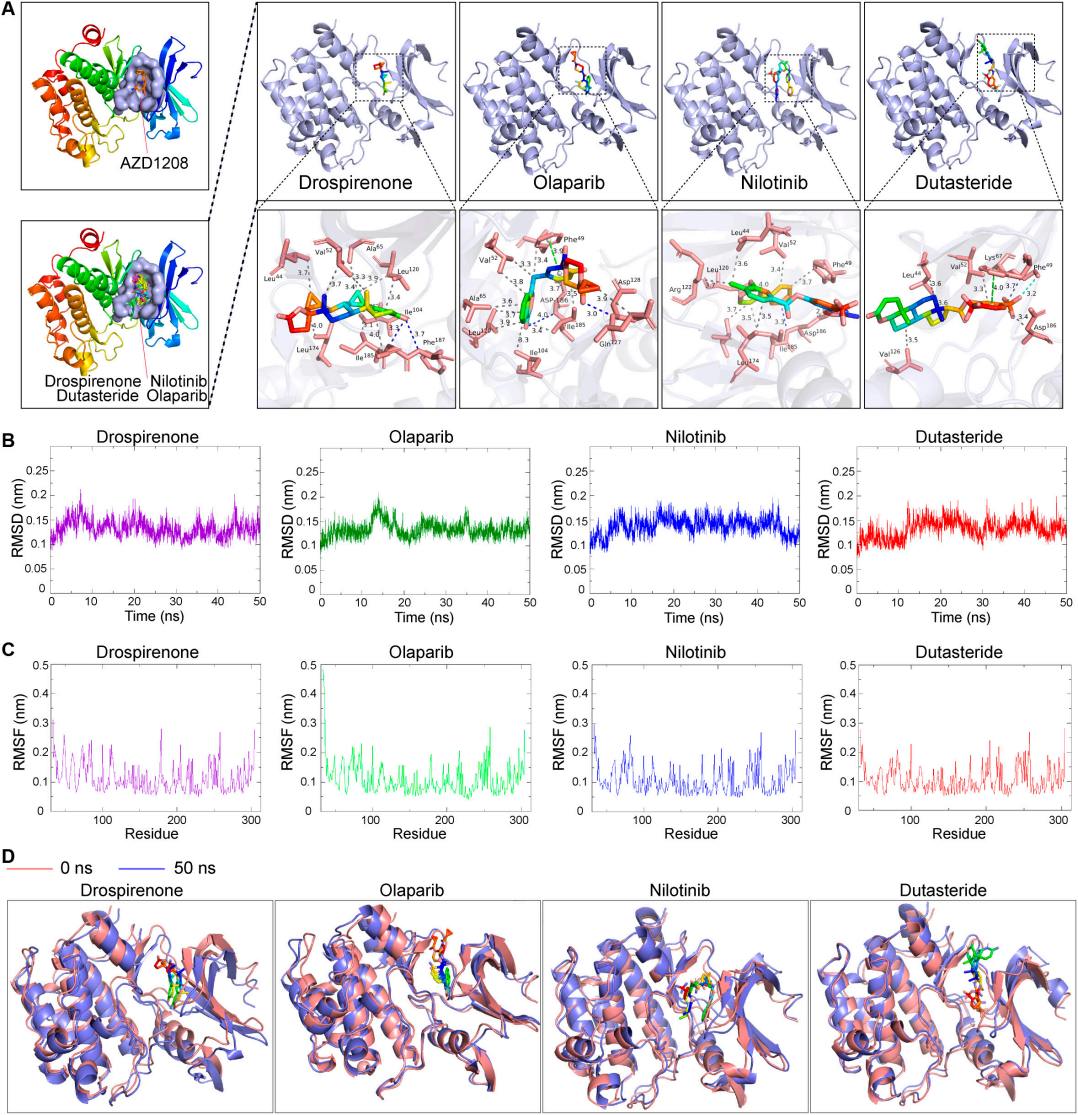
Molecular docking and molecular dynamics simulations of Pim1. (A) Molecular docking showing that drospirenone, olaparib, nilotinib, and dutasteride dock to Pim1 (PDB 1XWS) in the same drug-binding pocket as AZD1208 and that the bonds between the 4 drugs and Pim1. The blue dashed line represents hydrogen bonds, gray represents hydrophobic bonds, green cyan represents halogen bonds, and green represents π-stacking. (B) RMSD of 50-ns molecular dynamics simulations between Pim1 and drospirenone, olaparib, nilotinib, and dutasteride, respectively. (C) RMSFs of 50-ns molecular dynamics simulations between Pim1 and drospirenone, olaparib, nilotinib, and dutasteride, respectively. (D) The original and terminal structures of Pim1 interacting with drospirenone, olaparib, nilotinib, and dutasteride in 50-ns molecular dynamics simulations.

Pim1 was subjected to molecular docking and dynamic simulations to screen targeted drugs from the FDA Drugs Database. The drug-binding pocket of Pim1 with the highest drug score was the one to which AZD1208 was docked (Fig. 6A and Fig. S5A). The various structures of human Pim1 used for molecular docking were as follows: 1XWS, 2BIK, 2BZH, 5N4V, 6YKD, and Pim1-AF. The top 30 drugs with the lowest binding energy for each molecular docking with human Pim1 were intersected, and 4 drugs, namely, drospirenone, olaparib, nilotinib, and dutasteride, were screened out. In addition, in the molecular docking with mouse Pim1 (Protein Data Bank [PDB] Mpim1), nilotinib and olaparib were also ranked in the top 30, whereas dutasteride and drospirenone were ranked slightly lower. The binding energy and the rankings of the 4 drugs in each docking were listed in Table S1. Visual analysis revealed that drospirenone, olaparib, nilotinib, and dutasteride docked to human Pim1 at the same drug-binding pocket as AZD1208, through hydrogen bonds, hydrophobic bonds, halogen bonds, and π-stacking (Fig. 6A and Fig. S5B to F). The Pim1–drug complexes of the 4 drugs were stabilized during the 50-ns simulations, as indicated by the root mean square deviation (RMSD) (Fig. 6B). Simultaneously, the root mean square fluctuation (RMSF) indicated the fluctuations of Pim1 residues, which suggested that there were some conformational changes of the Pim1 structure to accommodate the drugs to create stable complexes (Fig. 6C). Furthermore, at the termination of the 4 simulations, the structures of the Pim1–drug complexes showed no notable changes compared with the original Pim1–drug complex structures, respectively (Fig. 6D). These results suggest that drospirenone, olaparib, nilotinib, and dutasteride may specifically bind to Pim1 and have therapeutic potential for the treatment of inflammatory arthritis.

### Nilotinib inhibits Th17 cell differentiation and alleviates inflammatory arthritis by targeting Pim1

**Fig. 7.**
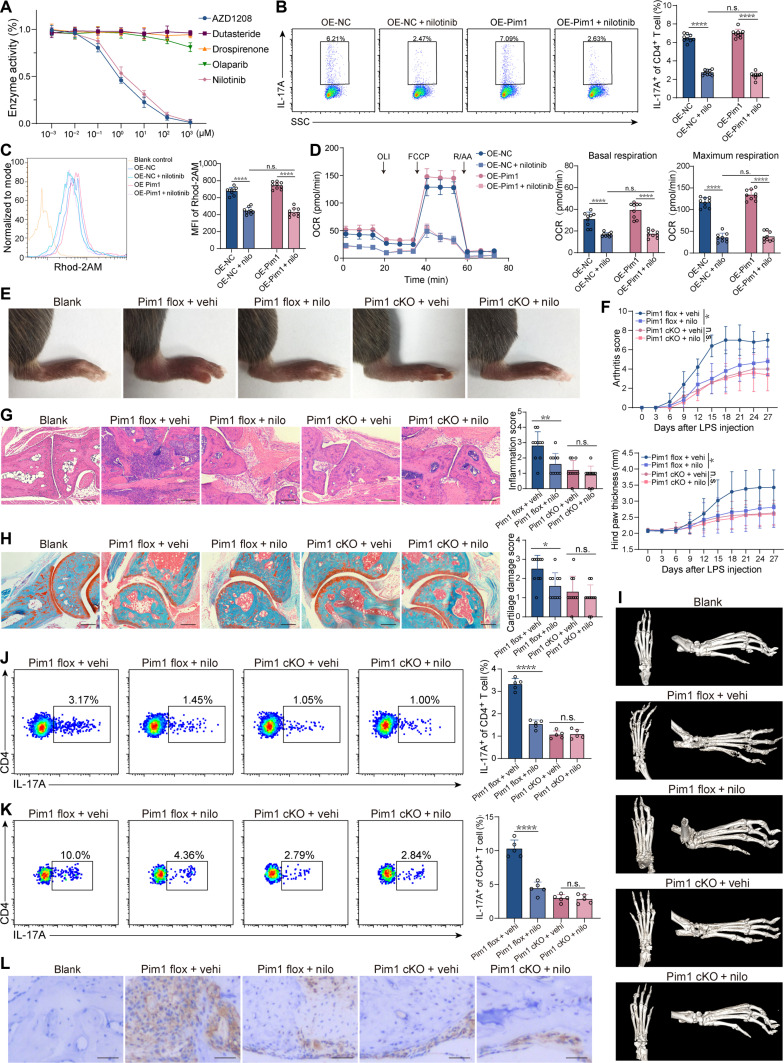
Nilotinib inhibits Th17 cell differentiation and alleviates inflammatory arthritis by targeting Pim1. (A) Enzyme activity of Pim1 after incubation with AZD1208, drospirenone, olaparib, nilotinib, or dutasteride (*n* = 9). (B) Frequency of Th17 cells among CD4^+^ cells after treatment with nilotinib in the presence or absence of Pim1 overexpression (*n* = 9). (C) MFI of Rhod-2AM in cells treated with nilotinib in the presence or absence of Pim1 overexpression (*n* = 9). (D) OCR of cells treated with nilotinib in the presence or absence of Pim1 overexpression (*n* = 9). (E) Macroscopic images of the ankles of Pim1 flox and Pim1 cKO CIA mice treated with vehicle or nilotinib. (F) Arthritis scores and hind paw thickness of Pim1 flox and Pim1 cKO CIA mice treated with vehicle or nilotinib (*n* = 5). (G and H) Representative histological images with H&E staining (G) and safranin O-fast green staining (H) of the ankles of Pim1 flox and Pim1 cKO CIA mice treated with vehicle or nilotinib (*n* = 10). Scale bars, 200 μm. (I) Representative micro-CT images of ankles of Pim1 flox and Pim1 cKO CIA mice treated with vehicle or nilotinib. (J and K) Frequency of Th17 cells among CD4^+^ cells in spleens (J) and ankles (K) of Pim1 flox and Pim1 cKO CIA mice treated with vehicle or nilotinib (*n* = 5). (L) Representative immunohistochemical images showing IL-17A expression in the ankle tissues of Pim1 flox and Pim1 cKO CIA mice treated with vehicle or nilotinib. Scale bars, 50 μm. The data were statistically analyzed via one-way ANOVA, followed by Bonferroni’s post hoc comparisons (B to D, G, H, J, and K) and 2-way repeated-measures ANOVA (F).

The results showed that nilotinib exhibited a concentration-dependent inhibitory effect on the enzyme activity of Pim1, although it was slightly weaker than that of AZD1208, whereas drospirenone, olaparib, and dutasteride did not affect Pim1 activity (Fig. 7A). In addition, the frequency of Th17 cells among CD4^+^ T cells was decreased to the same extent by nilotinib treatment with or without Pim1 overexpression (Fig. 7B). Similar effects were observed in the inhibited expression of RORγt, pSTAT3, and Th17-cell-associated pathogenic genes (Fig. S6A and B). However, drospirenone, olaparib, and dutasteride had no significant regulatory effect on the differentiation of Th17 cells (Fig. S6C). Furthermore, the increase in mito-Ca^2+^ concentration under Pim1 overexpression condition was decreased by nilotinib rather than drospirenone, olaparib, and dutasteride (Fig. 7C and Fig. S6D). Moreover, decreased mitochondrial potential, NADH and ATP levels, and basal and maximum OCR were observed following nilotinib treatment regardless of Pim1 overexpression (Fig. 7D and Fig. S6E to G). These results suggest that nilotinib inhibits Th17 cell differentiation by reducing the enzyme activity of Pim1.

In Pim1 flox CIA mice, nilotinib significantly alleviated swelling and redness as indicated by arthritis scores and hind paw thickness, which was not observed in Pim1 CD4-cre cKO mice (Fig. 7E and F). In addition, nilotinib reduced inflammatory infiltration, cartilage erosion, and bone destruction in Pim1 flox mice, whereas these effects were not observed in Pim1 cKO mice (Fig. 7G to I). In terms of the Th17 cell responses, nilotinib inhibited Th17 cell differentiation and reduced IL-17A expression in Pim1 flox mice but not in Pim1 cKO mice (Fig. 7J to L). These results suggest that nilotinib inhibits Th17 cell differentiation and inflammatory arthritis through targeting Pim1 in CD4^+^ cells.

**Fig. 8.**
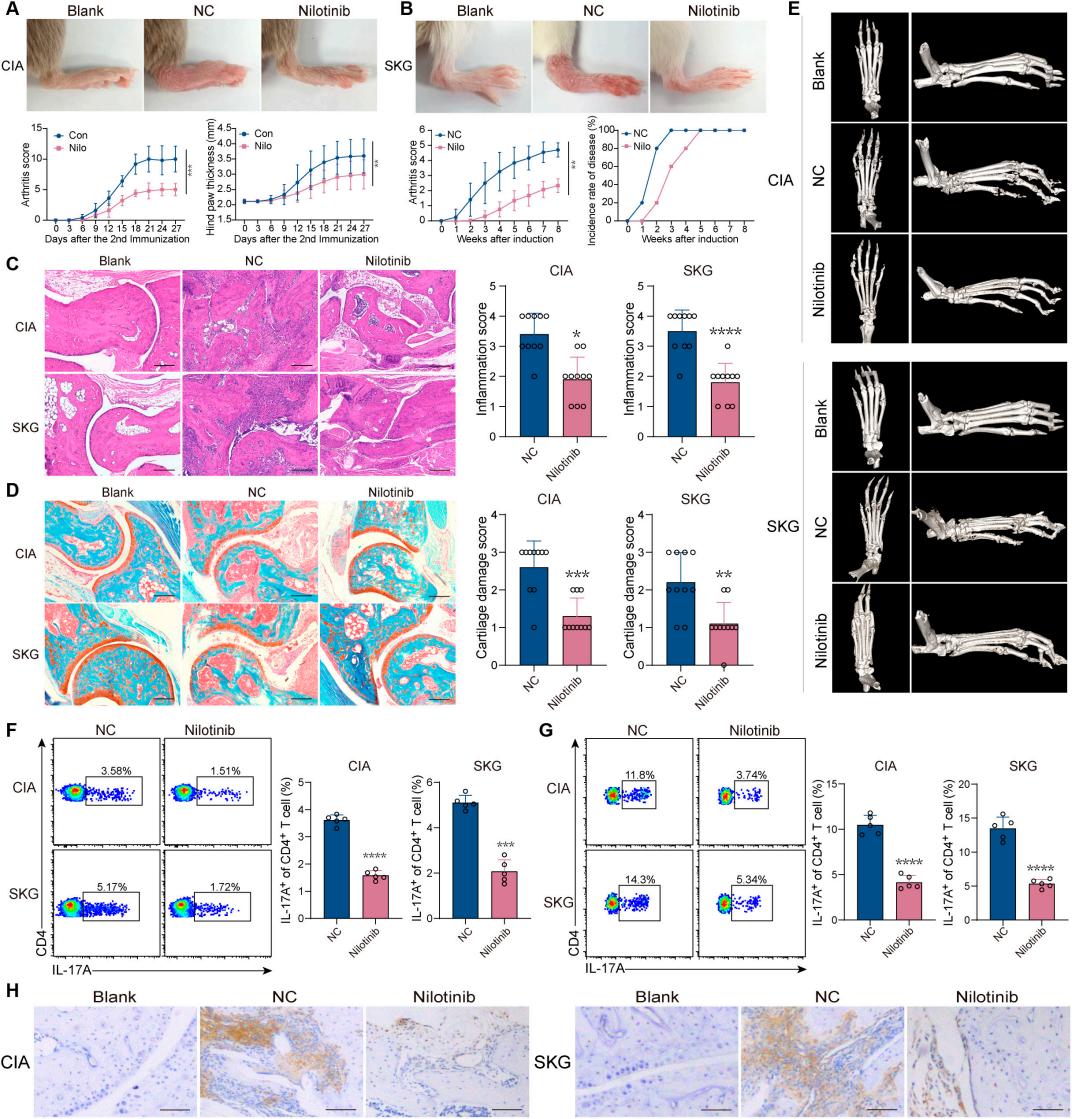
Nilotinib acts as an effective drug for the treatment of inflammatory arthritis. (A) Macroscopic images of the ankles, arthritis scores, and hind paw thickness of CIA mice treated with vehicle (NC group) and nilotinib (nilotinib group) (*n* = 5). (B) Macroscopic images of the ankles, arthritis scores, and incidence rate of SKG arthritis in the NC and nilotinib groups (*n* = 5). (C and D) Representative histological images with H&E staining (C) and safranin O-fast green staining (D) of the ankles of CIA and SKG arthritis mice in the NC and nilotinib groups (*n* = 10). Scale bars, 200 μm. (E) Representative micro-CT images of the ankles of CIA and SKG arthritis mice in the NC and nilotinib groups. (F and G) Frequency of Th17 cells among CD4^+^ cells in the spleens (F) and ankles (G) of CIA and SKG arthritis mice in the NC and nilotinib groups (*n* = 5). (H) Representative immunohistochemical images showing IL-17A expression in the ankle tissues of CIA and SKG arthritis mice in the NC and nilotinib groups. Scale bars, 50 μm. The data were statistically analyzed via 2-way repeated-measures ANOVA (A and B) and 2-tailed Student’s *t* test (C, D, F, and G).

The therapeutic effects of nilotinib were further verified in CIA and SKG mice. Arthritis manifestations and scores were alleviated by nilotinib in the CIA and SKG mice. CIA mice presented a smaller hind paw thickness, and SKG mice presented a slower onset of arthritis in nilotinib-treated group (Fig. 8A and B). Consistently, inflammatory infiltration, cartilage erosion, and bone destruction in CIA and SKG mice were improved by nilotinib (Fig. 8C to E). The Th17 cell frequency was decreased in CD4^+^ T cells in spleen and ankle joint tissues of CIA and SKG mice treated with nilotinib (Fig. 8F and G). The expression of IL-17A was also decreased in ankle tissues following nilotinib treatment (Fig. 8H). To confirm that nilotinib suppresses Th17 cell differentiation and inflammatory arthritis development via inhibiting Pim1 activity, we measured the expression of Pim1. Treatment with the Pim1 inhibitor AZD1208 did not alter Pim1 expression in differentiated Th17 cells in vitro or CD4^+^ T cells of CIA and SKG mice (Fig. S7A to C). Similarly, after nilotinib treatment, no significant change in the expression of Pim1 was observed both in vitro and in vivo (Fig. S7D to G). Furthermore, no significant change in the body weights of CIA and SKG arthritis mice was found during treatment with nilotinib (Fig. S8A). The counts of red blood cells (RBCs), white blood cells (WBCs), platelets (PLTs), the QT interval, and the left ventricular ejection fraction (LVEF) were not altered by nilotinib (Fig. S8B and C). In addition, there was no significant change in the serum alanine transaminase, alanine transaminase, total bilirubin, serum creatinine, urea nitrogen, serum amylase, or blood glucose levels after nilotinib treatment (Fig. S8D to F). These results suggest that nilotinib exerts its therapeutic effects with the suppressed Th17 cell response and is well tolerated in inflammatory arthritis.

## Discussion

Inflammatory arthritis is a group of chronic autoimmune diseases characterized by bone and joint destruction that seriously affects the health and the quality of life of patients [1,21,22]. During the development of inflammatory arthritis, a variety of proinflammatory cells contribute to the pathological processes, among which the abnormal Th17 cell differentiation is extensively involved in the joint inflammation, bone erosion, and cartilage destruction [2–4]. Previous studies have demonstrated that Pim1, as a key regulator of the biological activities of T cells, participates in the development of autoimmune uveitis by regulating T cell differentiation [23]. In CIA mice, Pim1 is also involved in regulating the differentiation of CD4^+^ T cell subsets [10]. In the present study, Pim1 expression was increased in the CD4^+^ T cells of patients with RA and AS as well as in those of the mice with inflammatory arthritis, both locally and systematically. Specifically intercepting Pim1 in CD4^+^ T cells prominently alleviated the development of inflammatory arthritis. These results indicate that the elevated expression of Pim1 in CD4^+^ T cells is greatly involved in the pathogenesis of inflammatory arthritis.

The therapeutic effects of targeting Pim1 have been extensively explored in autoimmune diseases such as lupus nephritis and autoimmune uveitis [23,24]. Previously, Pim1 inhibition was shown to exhibit a therapeutic effect on the cartilage erosion in the CIA model, whereas the detailed effects of Pim1 inhibition on the pathogenesis of inflammatory arthritis and the underlying mechanisms in vivo remain to be elucidated [10]. In the present study, we specifically inhibited Pim1 in CD4^+^ T cells via 2 methods, constructing Pim1 cKO mice and administering the Pim1 inhibitor AZD1208. In addition to confirming the improvement in cartilage erosion by Pim1 inhibition, the alleviation of inflammatory infiltration and bone destruction, 2 other important pathological features of inflammatory arthritis, was further verified in CIA mice, suggesting that Pim1 inhibition exhibits therapeutic effects on multiple pathological processes in inflammatory arthritis. These therapeutic effects were also observed in SKG mice, which constitute another animal model of inflammatory arthritis. In addition, the preventive administration of the Pim1 inhibitor AZD1208 also restrained the development of inflammatory arthritis. These results further validated Pim1 as an effective therapeutic target for treating inflammatory arthritis. In both CIA and SKG arthritis mice, Th17 cell differentiation of splenic CD4^+^ T cells and IL-17A expression in ankle tissues were decreased after the administration of the Pim1 inhibitor. In vitro experiments revealed that Pim1 inhibition blocked the expression of IL-17A, IL-17F, IL-26, and GM-CSF, which are crucial pathogenic cytokines for Th17 cells in exacerbating the pathological process of inflammatory arthritis [4]. These results further demonstrated that impeding Th17 cell differentiation was the key to Pim1 inhibition hindering the progression of inflammatory arthritis.

In addition to its role in autoimmune diseases, Pim1 has also been broadly studied as a therapeutic target in T cell tumors, breast cancer, and Alzheimer’s disease [6,7,25]. However, the Pim1 inhibitor AZD1208 remains unapproved for the clinical treatment of human diseases because of the high incidence of adverse events observed in a phase I clinical trial [26]. To screen clinically approved drugs that can specifically bind to Pim1 and inhibit its activity, Pim1 was subjected to molecular docking and molecular dynamics simulations, thereby identifying nilotinib. Nilotinib was previously found to affect Th17 cell differentiation, but the detailed mechanism was unclear [27]. In the present study, we provided further findings that nilotinib restrained the inflammatory infiltration and bone and cartilage destruction of inflammatory arthritis, together with inhibition of Th17 cell differentiation and IL-17A expression. However, these effects were blocked in inflammatory arthritis mice with specific knockout of Pim1 in CD4^+^ cells, suggesting that nilotinib exerts its therapeutic effects by impeding the promotive effect of Pim1 on Th17 cell differentiation. Nilotinib is originally a chemotherapy drug, and its side effects on cardiac, hematopoietic, hepatic, renal, and pancreatic functions are of great concern [28–30]. However, in our study, nilotinib did not cause the significant side effects mentioned above during CIA and SKG arthritis treatment at the administered dose. The differences in side effects between our findings and those of previous studies might be related to the dosage of nilotinib used. The dose of nilotinib used to treat inflammatory arthritis mice in our study was much lower than that used as a chemotherapy drug in human patients, as calculated by interspecies dose translation [31]. These results suggest that nilotinib might be an effective and safe substitute for the currently clinically nonapproved Pim1 inhibitors to treat inflammatory arthritis, and long-term toxicology studies in relevant preclinical models should be conducted before clinical used.

Metabolism encompasses carbohydrate metabolism, lipid metabolism, and amino acid metabolism, which participate in regulating the differentiation of various immune cells, including Th17 cells, thereby contributing to the pathogenesis of autoimmune diseases [4,11,32]. During Th17 cell differentiation, significant metabolic reprogramming occurs, with enhanced glycolysis, OXPHOS, and fatty acid synthesis, which yielded identical results in the present study [33–35]. However, Pim1 inhibition disturbed OXPHOS but had no significant inhibitory effect on glycolysis or fatty acid synthesis, suggesting that Pim1 promotes Th17 cell differentiation through enhancing OXPHOS activity. A previous study revealed that Th17 cells rely more on glycolysis for the rapid energy production [36,37]. However, in the present study, the increase in OXPHOS activity coincided with increased ATP production during Th17 cell differentiation, whereas inhibiting OXPHOS resulted in the inhibition of Th17 cell differentiation with a concomitant decrease in ATP production, suggesting that OXPHOS plays an indispensable role in the energy supply and that a new equilibrium may be reached between OXPHOS and glycolysis during the Th17 cell differentiation process. Furthermore, OXPHOS is essential for the activation of the T cell receptor, mammalian target of rapamycin, and basic leucine zipper ATF-like transcription factor (BATF) pathways, and the production of IL-17A, IL-17F, and GM-CSF, all of which promote the differentiation and the proinflammatory functions of Th17 cells [12,13,35]. In addition, OXPHOS induces resistance to apoptosis and promotes the persistence of Th17 cells by inhibiting the activity of apoptotic factors [38]. These results suggest that OXPHOS, in addition to providing energy, exerts multiple physiological functions distinct from those of glycolysis, which are important during the differentiation of Th17 cells.

Pim1 participates in regulating the homeostasis of intracellular Ca^2+^ including mito-Ca^2+^, which is essential for the metabolic activity of OXPHOS and the maintenance of the functional state of T cells [16,39–41]. We found that Pim1 promoted mito-Ca^2+^ uptake, thereby enhancing the metabolic activity of OXPHOS. However, during Th17 cell differentiation, the cellular uptake of extracellular Ca^2+^ and the release of Ca^2+^ from the endoplasmic reticulum rapidly increased, resulting in an increased source of mito-Ca^2+^ [18,42]. Excessive mito-Ca^2+^ influx may cause mito-Ca^2+^ overload and disruption of mitochondrial structure and OXPHOS [43]. In the present study, Pim1 overexpression further promoted mito-Ca^2+^ influx along with the enhanced OXPHOS activity. In addition, Pim1 overexpression exhibited a preventive effect against mito-Ca^2+^ overload-induced damage to the mitochondrial ultrastructure [16]. These results demonstrated that the promotion of mito-Ca^2+^ influx by Pim1 is exquisitely regulated in a limited and controllable manner to fine-tune mitochondrial OXPHOS activity. Mito-Ca^2+^ influx is tightly regulated by protein phosphorylation. The intracellular Ca^2+^ concentration and its transport profile are manipulated by protein phosphorylation [44,45]. Furthermore, the activity of the mitochondrial calcium uniporter protein (MCU) is promoted by phosphorylation [46]. As a serine/threonine phosphokinase, Pim1 regulates intracellular Ca^2+^ homeostasis through its phosphorylation function [39,47]. We showed that Pim1 interacted with MICU1 and increased the phosphorylation level of MICU1. Previously, another phosphokinase AKT was found to inhibit MCU activity by phosphorylating MICU1 [20], indicating that Pim1 may promote mito-Ca^2+^ uptake by mediating the phosphorylation of MICU1.

Limitations of this study should be acknowledged. First, the detailed phosphorylation site of MICU1 and the mechanism of the Pim1/MICU complex remain unclear. Future study should identify the phosphorylation sites of MICU1 and conduct functional validation to determine the key sites modified by Pim1 that regulate mito-Ca^2+^ influx. Second, Pim1 promotes the pathogenic functions of CD8^+^ T cells and synovial fibroblasts, which are involved in the progression of inflammatory arthritis [48–50]. The possibility that Pim1 inhibition exerts its therapeutic effects through inhibiting the functions of these pathogenic cells cannot be ruled out. Adoptive transfer of AZD1208- or nilotinib-treated CD4^+^ T cells to CIA or SKG mice deficient in CD4^+^ T cells, or specific delivery of AZD1208 or nilotinib to CD4^+^ T cells, will help overcome the limitation of the nontargeted effects of AZD1208 and nilotinib in vivo. Finally, in the treatment of inflammatory arthritis with nilotinib, the toxicity monitoring based on short-term hematological and serum biochemical parameters is insufficient to form a comprehensive assessment of the safety of nilotinib. More rigorous and extensive long-term toxicological studies must be conducted in preclinical models before clinical consideration. In conclusion, our study uncovers the mechanism through which Pim1 promotes the differentiation of Th17 cells in inflammatory arthritis and advances the clinical application of Pim1 as a therapeutic target in the treatment of inflammatory arthritis.

## Materials and Methods

### Study approval

The animal research experiments were approved by the Institutional Animal Care and Use Committee of Sun Yat-Sen University (approval number: 2022002464). The experiments with human samples were approved by the Ethics Committee of the Eighth Affiliated Hospital, Sun Yat-Sen University (approval number: 2024r039). The clinical characteristics of the patients with RA and AS and their controls are shown in Tables S2 and S3.

### Mice

Pim1 flox and Pim1 cKO mice were purchased from Shanghai Model Organism. DBA/1 mice were purchased from GemPharmatech. SKG mice were gifts from Shanghai Model Organism. All animals in this study were maintained in a specific-pathogen-free barrier facility.

### CIA model

CIA model was induced in DBA/1 mice and scored as previously described [51]. For the induction of CIA in gene-edited mice with a C57BL/6 background, Freund’s complete adjuvant (Chondrex, #7023) was used, and 35 μg of lipopolysaccharide (Sigma-Aldrich, #L2880) was injected intraperitoneally 4 weeks after the second immunization. Hind paw thickness was measured with a vernier calipers.

### SKG arthritis model

SKG arthritis model was induced and scored as previously described [52].

### Identification of gene-edited mice

DNA was extracted and then amplified using forward primer TCGGCTAGCTTTCCGTGGTA and reverse primer TGATCCCCTCGGTCCATGTC to determine the genotype of Pim1. The wild-type-Pim1 band was detected at 301 bp, and the Pim1 flox band was detected at 355 bp. The CD4-cre sequence was amplified using forward primer CATGTCCATCAGGTTCTTGC and reverse primer CCAGGGTCGGAGACAATAAC, and the CD4-cre band was detected at 500 bp.

### Drug administration in vivo

For the prevention of CIA, AZD1208 (1 mg/ml; Selleck, #S7104; 0.5% methylcellulose and 0.1% Tween 80 in water) was orally administered at a dose of 10 mg/kg once a day from day 1 to day 21. For the treatment of CIA, AZD1208 (1 mg/ml) or nilotinib (1 mg/ml; Aladdin, #N126111; 0.5% methylcellulose in water) was administered at the doses of 10 and 5 mg/kg, respectively, from day 21 to day 48. For the treatment of CIA in gene-edited mice, nilotinib was administered from day 48 to day 75. For the prevention of SKG arthritis, AZD1208 (1 mg/ml) was orally administered at a dose of 10 mg/kg once a day for 4 weeks before arthritis induction. For the treatment of SKG arthritis, AZD1208 (1 mg/ml) or nilotinib (1 mg/ml) was administered once a day at doses of 10 and 5 mg/kg, respectively, for 8 weeks after arthritis induction.

### Mouse CD4^+^ T cells and F4/80^+^ macrophage purification

The mouse ankle joints were dissected and digested with 0.5% type II collagenase (Gibco, #17101-015). The discrete cells were collected, and the CD4^+^ T cells and F4/80^+^ macrophages were sorted by flow cytometry using anti-CD4–phycoerythrin (PE) (BioLegend, #100407) or anti-F4/80–PE (BioLegend, #111603), respectively. The splenic CD4^+^ T cells were sorted via flow cytometry as described above.

### Human CD4^+^ T cell purification

Human peripheral blood mononuclear cells were extracted and CD4^+^ T cells were purified via anti-CD4 microbeads (Miltenyi Biotec, #130-045-101) or a naïve CD4^+^ T cell isolation kit (Miltenyi Biotec, #130-094-131).

### Naïve CD4^+^ T cell polarization

Naïve CD4^+^ T cells were treated with anti-CD3 (1 μg/ml; BioGems, #05121-25-500), anti-CD28 (1 μg/ml; BioGems, #10311-25-500), and IL-2 (10 U/ml; Prosperich, #CSBSJ-IL-2). For Th1 cell polarization, the cells were treated with IL-12 (10 ng/ml; Peprotech, #200-12) and anti-IL-4 (10 μg/ml; BioGems, #81121-25). For Th2 cell polarization, the cells were treated with IL-4 (5 ng/ml; Peprotech, #200-04) and anti-interferon-γ (IFN-γ; 10 μg/ml; BioGems, #80821-25). For Th17 cell polarization, the cells were treated with transforming growth factor-β (TGF-β; 10 ng/ml; Peprotech, #100-21C), IL-6 (10 ng/ml; Peprotech, #200-06), IL-23 (20 ng/ml; Peprotech, #200-23), anti-IFN-γ (10 μg/ml), and anti-IL-4 (10 μg/ml). For Treg cell polarization, the cells were supplemented with TGF-β (2.5 ng/ml). For the indicated experiments, AZD1208 (10 μM), CCCP (5 μM), RR (5 μM), Ru360 (2 μM), and nilotinib (5 μM) were added. The cells were polarized for 5 d.

### Flow cytometry analysis

For analysis of human Th1, Th2, and Th17 cells, cell stimulation and protein transport inhibitor cocktail (Invitrogen, #00-4975-03) was added 6 h before collection. The cells were stained with live/dead violet dye (BioLegend, #423113), fixed, permeabilized, and then stained with anti-IFN-γ–APC (BioLegend, #986702), anti-IL-4–APC (BioLegend, #500811), anti-IL-17A–Alexa Fluor 647 (BD Biosciences, #560437), and anti-Foxp3–Alexa Fluor 647 (BioLegend, #320014), respectively. The cells were analyzed with a BD FACSCelesta cytometer.

Mouse splenocytes were stained with live/dead violet dye and anti-CD4–PE (BioLegend, #100407). After fixation and permeabilization, the cells were stained with anti-IFN-γ–APC (BioLegend, #505809), anti-IL-4–APC (BioLegend, #504105), anti-IL-17A–APC (BioLegend, #506915), and anti-Foxp3–Alexa Fluor 647 (BioLegend, #320013) for 30 min, respectively.

For analysis of Th17 cell differentiation of CD4^+^ T cells in joint tissues, the dissected ankle joint tissue cells were harvested as described above. The cells were stained with live/dead violet dye, anti-CD4–PE, and IL-17A–APC.

The cells were stained with 100 nM TMRE (Beyotime, #C2001S) for detection of the mitochondrial membrane potential, stained with 5 μM Rhod-2AM (Abcam, #ab142780) for detection of mito-Ca^2+^, and stained with 10 μM 2′,7′-dichlorodihydrofluorescein diacetate (DCFH-DA; Beyotime, #S1105S) for the detection of ROS.

### Lentivirus and plasmid transfection

Human Pim1-overexpressing and MICU1-silencing lentivirus and human Pim1- and MICU1-overexpressing plasmids were constructed by OBiO Technology. For the transfection of human CD4^+^ T cells, lentivirus was added at a multiplicity of infection of 75 together with polybrene (5 μg/ml). For the transfection of human embryonic kidney (HEK) 293T cells, the plasmid and Lipofectamine 3000 reagent (Invitrogen, #L3000015) were mixed and incubated at 37 °C for 20 min. The mixture was then added to the cells.

### RNA preparation and RT-qPCR

RNA was prepared and RT-qPCR were performed via the RNA Quick Purification Kit (ES Science, #RN001), the RT Master Mix (AG, #AG11706), and the SYBR Green Premix Kit (AG, #AG11718). Gene expression was quantified via the 2^−ΔΔCt^ method and normalized to hypoxanthine-guanine phosphoribosyltransferase 1 (Hprt1). The primer sequences used in this study are shown in Table S4.

### Western blot analysis

IP and Western blot were performed and analyzed as previously described [51]. The following antibodies were used: anti-PIM1 (mouse: Abcam, #ab308006; human: Cell Signaling Technology [CST], #54523S), anti-RORγt (Medical & Biological Laboratories, #M219-3), anti-STAT3 (Abcam, #ab68153), anti-pSTAT3 (CST, #9145), anti-β-ACTIN (Abcam, #ab8226), anti-MICU1 (CST, #12524S), anti-His (CST, #2365S), anti-Flag (CST, #14793S), anti-pan-phospho-serine/threonine (ABclonal, #AP1067), secondary anti-mouse immunoglobulin G (IgG; Boster, #BA1050), secondary anti-rabbit IgG (Boster, #BA1054), and control rabbit IgG for IP (Beyotime, #A7016).

### Enzyme-linked immunosorbent assay

Human IL-17A enzyme-linked immunosorbent assay (ELISA) kit (Elabscience, #E-EL-H5812), human IL-17F ELISA kit (Elabscience, #E-EL-H4193), human IL-22 ELISA kit (Elabscience, #E-EL-H0106), and human GM-CSF ELISA kit (Elabscience, #E-EL-H0081) were used. The cell supernatant was collected and added into the ELISA test plates. The biotinylated antibody working solution was added and incubated at 37 °C for 1 h. The samples were washed, added with horseradish-peroxidase-conjugated working solution, and incubated at 37 °C for 30 min. Then, the substrate solution was added and incubated at 37 °C for 15 min. The stop buffer was added, and the absorbance at 450 nm was measured using a luminometer.

### RNA sequencing and data analysis

RNA sequencing was performed, the clean reads were mapped to GCF_000001405.38_GRCh38.p12, and the data were analyzed via HISAT (v2.0.4), Bowtie (v2.2.5), DESeq2 (v1.4.5), and Dr. Tom (BGI). The differentially expressed genes were identified with a fold change cutoff of 2 and an adjusted *P* value of 0.05.

### ATP measurement assay

An ATP assay kit (Beyotime, #S0026) was used to measure ATP levels. The cells lysis supernatant was mixed with ATP detection buffer. ATP levels were measured via a luminometer and were presented as relative luminescence normalized to the protein concentration.

### NADH measurement assay

An NADH assay kit (Beyotime, #S0175) was used to measure NADH levels. The absorbance at 450 nm was measured via a luminometer, and the NADH levels were presented as the relative absorbance normalized to the protein concentration.

### OCR and extracellular acidification rate measurements

Seahorse XF Cell Mito Stress Test Kit (Seahorse, #103015-100) and Cell Glycolytic Stress Test Kit (Seahorse, #103020-100) were used to measure the OCR and extracellular acidification rate (ECAR), respectively. Equivalent numbers of cells were seeded and stuck to the bottom of an XF96 plate coated with Cell Tak reagent (Corning, #354240). The cells were loaded into the Seahorse XFe96 analyzer and subjected to the Mito stress or glycolytic stress. The OCR was calculated after sequential addition of oligomycin (1 μM), carbonyl cyanide *p*-trifluoromethoxyphenylhydrazone (FCCP; 1.5 μM), and rotenone/antimycin A (150 nM), and the ECAR was calculated after sequential addition of glucose (10 mM), oligomycin (1μM), and 2-deoxyglucose (50 mM).

### Electron microscopy

Cells were harvested and fixed in electron microscopy fixative solution (Thermo Fisher Scientific, #ED-8484). After fixation with osmium tetroxide and dehydration, the cells were counterstained with 3% uranyl acetate–lead citrate. Images were obtained using a transmission electron microscope system (Hitachi).

### Pim1 activity measurement

Pim1 activity was measured via a Pim1 kinase assay kit (Promega, #V4032 and #V6930) according to the manufacturer’s instructions and presented as relative luminescence.

### Molecular docking

Different PDB structures of human Pim1 (1XWS, 2BIK, 2BZH, 5N4V, 6YKD, and Pim1-AF) and Mpim1 were retrieved from the RCSB PDB (www.rcsb.org). The heteroatoms and the cocrystallized ligands were removed using PyMOL (2.5.3). All water molecules were removed, and hydrogen atoms were added to the PDB structures via AutoDockTools (1.5.7). Structures of FDA-approved molecules in mol2 format were downloaded from the ZINC website (zinc.docking.org) and transferred to PDB format via Openbabel (3.3.1). The dockings were then performed, and the binding energy was calculated via AutoDockTools. The lowest binding energy of each drug was selected out for ranking. The bonds between Pim1 and the drugs formed during docking were analyzed via the PLIP website (plip-tool.biotec.tu-dresden.de).

### Molecular dynamics simulation

Water molecules and the cocrystallized ligands in the PDB structure 1XWS of Pim1 were removed via PyMOL, and the missing atoms were replenished via SPDBV (4.1.0). The dynamic simulation was performed in the AMBER99SB ILDN protein force field via Gromacs (2022.2). The structure of Pim1 was transferred to a topological format, and the structural information of the ligand was added. The simulation box was constructed, and the minimum distance from the molecule to the edge of the box was set to 1.2 nm. The energy of the protein molecules in a vacuum was minimized, followed by filling of the box with solvents and ions and minimizing their energy. After a position-limited preequilibrium simulation, a 50-ns simulation was performed. The RMSD was calculated via g_rms and g_rmsdist, and the RMSF was calculated via g_rmsf.

### Micro-computed-tomography scanning

The samples were scanned via a Siemens Inveon computed tomography (CT) scanner. Three-dimensional image reconstruction was performed via the RadiAnt DICOM Viewer (v5.0.2).

### Histological staining

Hematoxylin and eosin (H&E) staining, safranin O-fast green staining immunofluorescence staining, and immunohistochemical staining were performed and scored as previously described [51]. The following antibodies were used: anti-CD4 (Signalway Antibody, #44038), anti-PIM1 (Bioss, #bs-3540R), anti-CD68 (Abcam, #ab955), anti-F4/80 (Santa Cruz Biotechnology, #sc-377009), secondary goat anti-rabbit IgG antibody (Invitrogen, #A-11008), secondary goat anti-mouse IgG antibody (Invitrogen, #A-21422), and anti-IL-17A (ABclonal, #A12454).

### Measurement of QT interval and LVEF

The mice were anesthetized using isoflurane. The electrodes were connected as indicated, and the electrocardiograms of the mice were obtained via a Mouse Monitor S (INDUS). The QT and RR intervals were measured, and the corrected QT interval was calculated. The LVEF was measured via an ultrasound real-time imaging system (VEVO 2100).

### Testing of blood samples

RBC, WBC, and PLT counts, hepatic functions, renal functions, the activity of serum amylase, and the concentration of blood glucose were detected by Huateng Bioscience.

### Statistical analysis

The data in this study are presented as histograms with scatter plots and means and SDs. Statistical analyses were performed via GraphPad Prism (8.0). The statistical methods used were stated in the corresponding figure legends. *P < 0.05, **P < 0.01, ***P < 0.001, and ****P < 0.0001 in the figures indicate statistically significant, and n.s. indicates no statistically significant.

## Ethical Approval

The animal experimental protocols were approved by the Institutional Animal Care and Use Committee of Sun Yat-Sen University (approval number: 2022002464). The collection of human samples and the experiments with human samples were approved by the Ethics Committee of the Eighth Affiliated Hospital, Sun Yat-Sen University (approval number: 2024r039).

## Data Availability

The RNA sequencing data have been deposited into the NCBI GEO database under GSE261671. All other data are available in the main text or the Supplementary Materials.
