# Supplementary material for: Pim1 Serves as a Therapeutic Target for Inflammatory Arthritis via Mitochondrial Metabolism and Th17 Cell Differentiation
Source: Research (Wash D C). 2026 Feb 27;9:1137. doi: 10.34133/research.1137 (PMC12946387; doi:10.34133/research.1137)
Supplement: Supplementary 1 — Figs. S1 to S8 Tables S1 to S4 Data files S1 to S3 [file research.1137.f1.zip › Supplemental material.docx]

Supplementary materials for

**Pim1 Serves as a Therapeutic Target for Inflammatory Arthritis via Mitochondrial Metabolism and Differentiation of Th17 Cell**

**This file includes:**

**Supplementary Figure 1** Pim1 expression in macrophages in human inflammatory arthritis and its mouse models

**Supplementary Figure 2** Pim1 acts as a therapeutic target in inflammatory arthritis

**Supplementary Figure 3** OXPHOS may participate in Pim1-regulated Th17 cell differentiation

**Supplementary Figure 4** Pim1 promotes mito-Ca2+, OXPHOS and Th17 cell differentiation through MICU1

**Supplementary Figure 5** Molecular docking of drugs with different PDB structures of Pim1

**Supplementary Figure 6** Effects of drugs on Th17 cell differentiation and OXPHOS

**Supplementary Figure 7** Effects of AZD1208 and Nilotinib on the expression of Pim1 in CD4+ T cells

**Supplementary Figure 8** Nilotinib exhibited no significant side effects during the treatment of inflammatory arthritis

**Supplementary Table 1** Binding energy and ranking of drugs in dockings with different PDB structures of Pim1

**Supplementary Table 2** Clinical characteristics of RA patients and controls

**Supplementary Table 3** Clinical characteristics of AS patients and controls

**Supplementary Table 4** Primer sequences used in this study

**Other Supplementary data for this manuscript includes the following:**

**Data file 1:** Gene expression of cells in the naïve group, Th17 group and AZD1208 group in the RNA sequencing.

**Data file 2:** Mass spectrometry data of the immunoprecipitates of Pim1.

**Data file 3:** Molecular docking data of Pim1 with FDA-approved drugs.

**
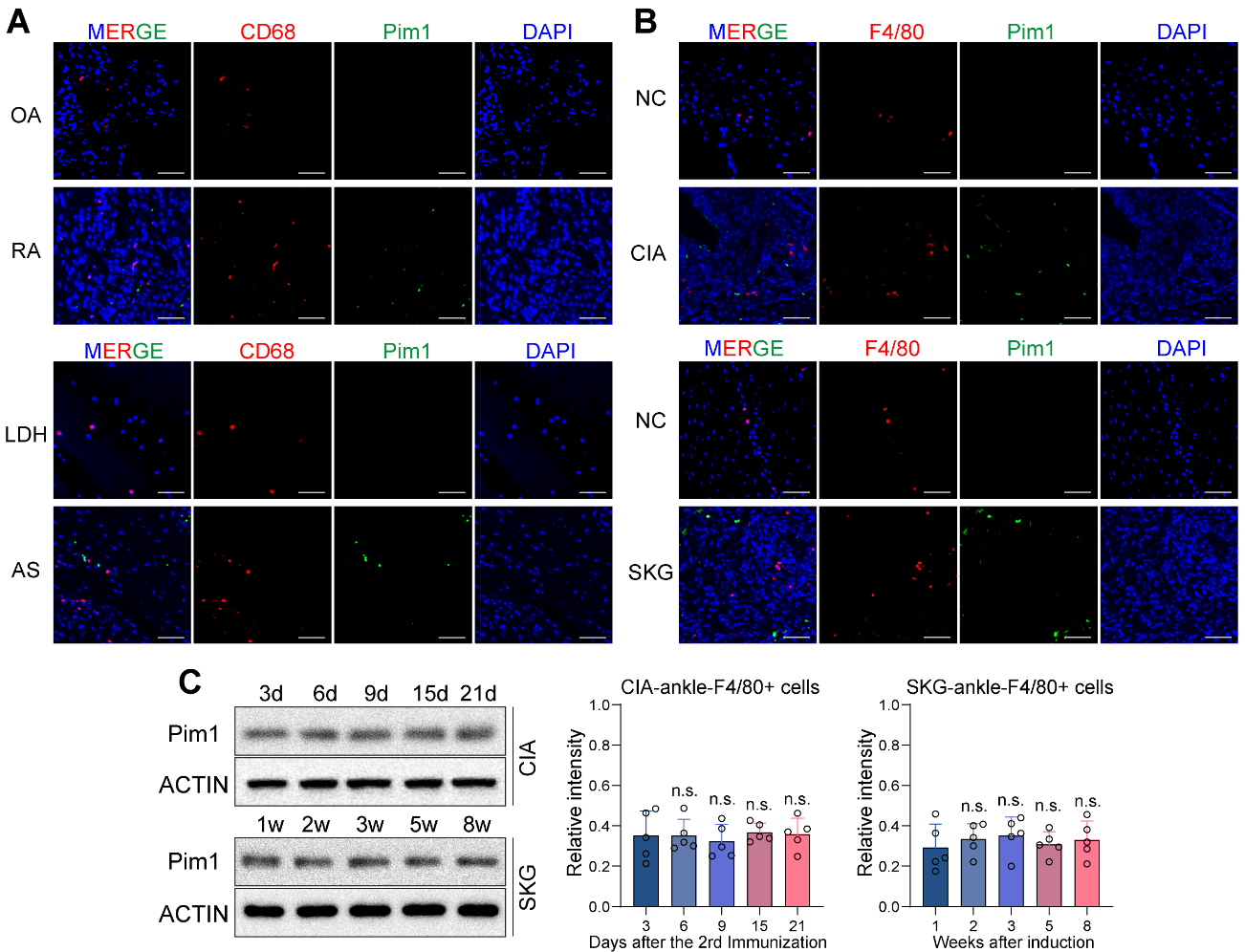
Supplementary Figure 1.** **Pim1 expression in macrophages in human inflammatory arthritis and its mouse models**

**A.** Representative IF images showing Pim1 expression in CD68+ cells in OA and RA synovium and LDH and AS enthesis. Scale bar = 50 μm. **B.** Representative IF images showing Pim1 expression in F4/80+ cells in ankle tissues from CIA mice and SKG arthritis mice and the corresponding normal controls. Scale bar = 50 μm. **C.** Relative protein levels of Pim1 in F4/80+ isolated from ankle tissues of CIA and SKG arthritis mice over time after arthritis induction (n = 5). The statistical analyses were performed as follows: One-way ANOVA followed by Bonferroni’s post hoc comparisons test (C).


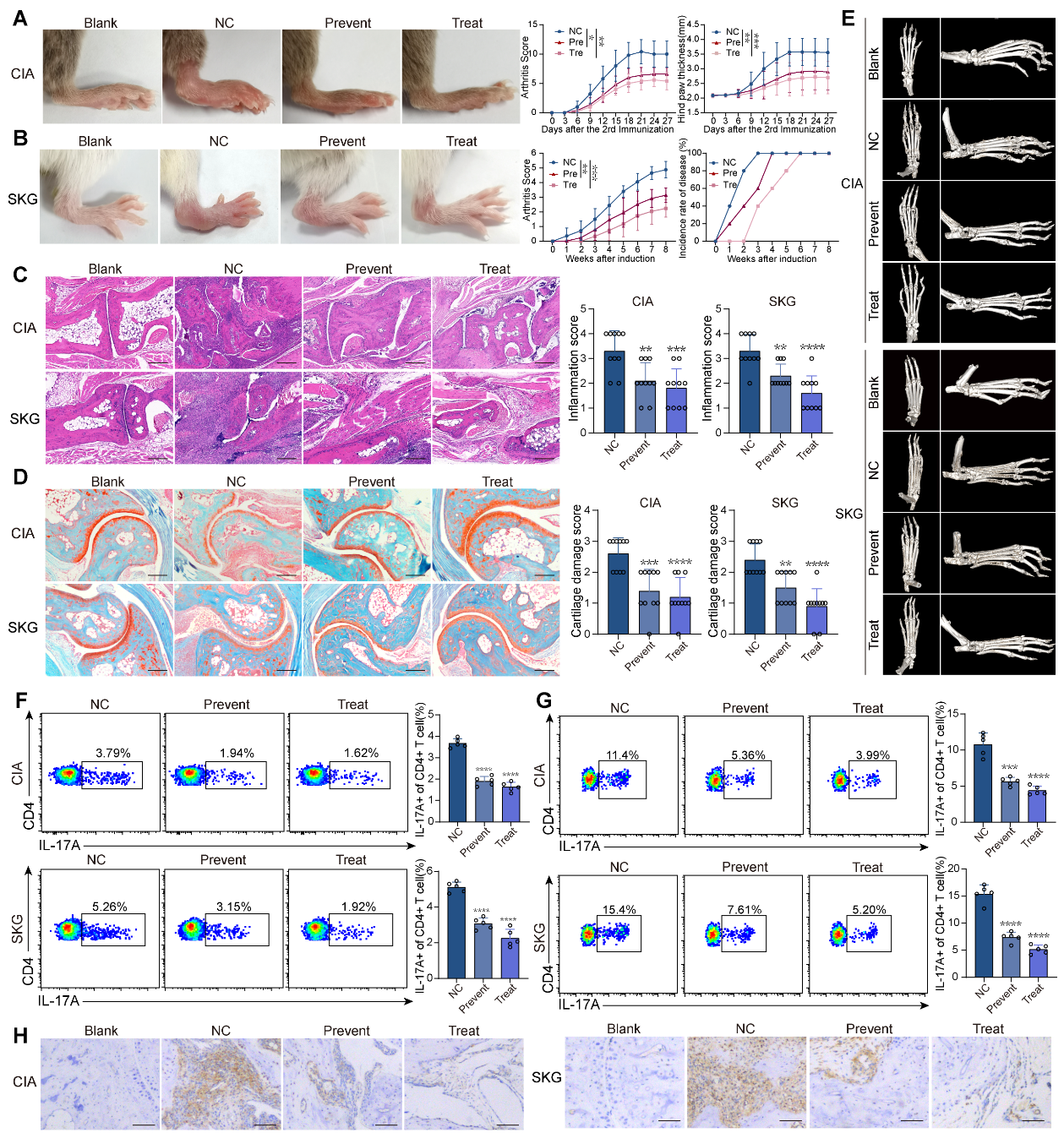
**Supplementary** **Figure 2.** **Pim1 acts as a therapeutic target in inflammatory arthritis**

**A.** Macroscopic images of ankles, arthritis scores and hind paw thickness of CIA mice in the NC, PRE and TRE groups (n = 5). **B.** Macroscopic images of ankles, arthritis scores and incidence rate of SKG arthritis mice in the NC, PRE and TRE groups (n = 5). **C-D.** Representative histological images with H&E staining (C) and safranin O-fast green staining (D) of the ankles of CIA and SKG arthritis mice in the NC, PRE and TRE groups (n = 10). Scale bar = 200 μm. **E.** Representative micro-CT images of ankles of CIA and SKG arthritis mice in the NC, PRE and TRE groups. **F-G.** Frequencies of IL-17A+ cells in CD4+ cells in spleens (F) and ankles (G) of CIA and SKG arthritis mice in the NC, PRE and TRE groups (n = 5). **H.** Representative IHC images showing IL-17A expression in the ankle tissues of CIA and SKG arthritis mice in the NC, PRE and TRE groups. Scale bar = 50 μm. The statistical analyses were performed as follows: two-way repeated-measures ANOVA (A, B) and one-way ANOVA followed by Bonferroni’s post hoc comparisons test (C, D, F, G).


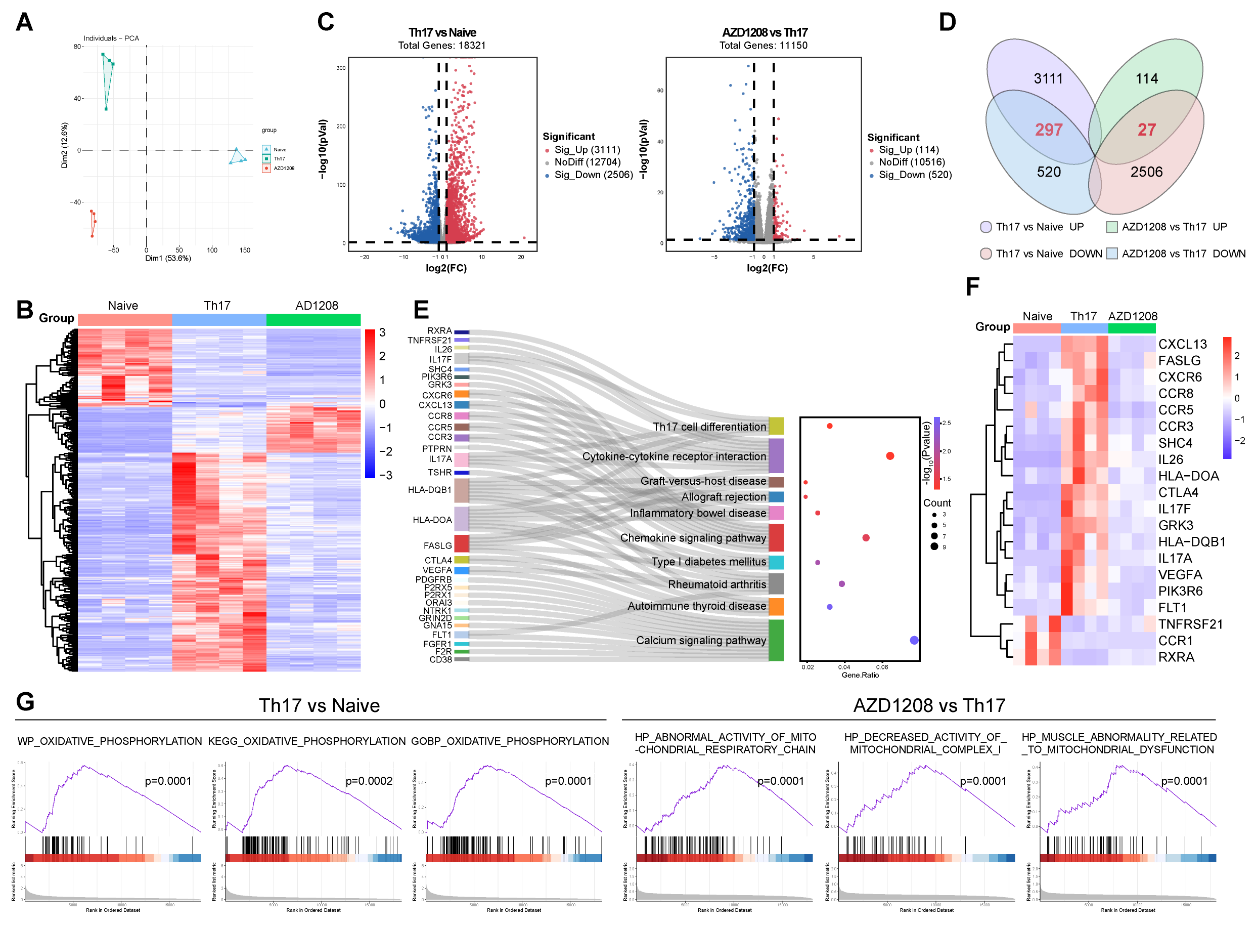


**Supplementary Figure 3. OXPHOS may participate in Pim1-regulated Th17** **cell differentiation**

**A.** Principal component analysis of RNA-seq of the Naïve, Th17 and AZD1208 groups (n = 4). **B.** Cluster heatmap of differentially expressed genes in the RNA-seq of the Naïve, Th17 and AZD1208 groups (n = 4). **C.** RNA-seq Volcano plots of the Naïve group versus the Th17 group and the Th17 group versus the AZD1208 group (n = 4). **D.** Venn diagram showing the number of Pim1-regulated Th17 cell differentiation-associated genes. **E.** GO-BP analysis of Pim1-regulated Th17 cell differentiation-associated genes enriched autoimmunity-related pathways (n = 4). **F.** Cluster heatmap of Pim1-regulated Th17 cell differentiation-associated genes that were related to autoimmunity (n = 4). **G.** GSEA of the Th17 group versus the Naïve group and the AZD1208 group versus the Th17 group (n = 4).

**
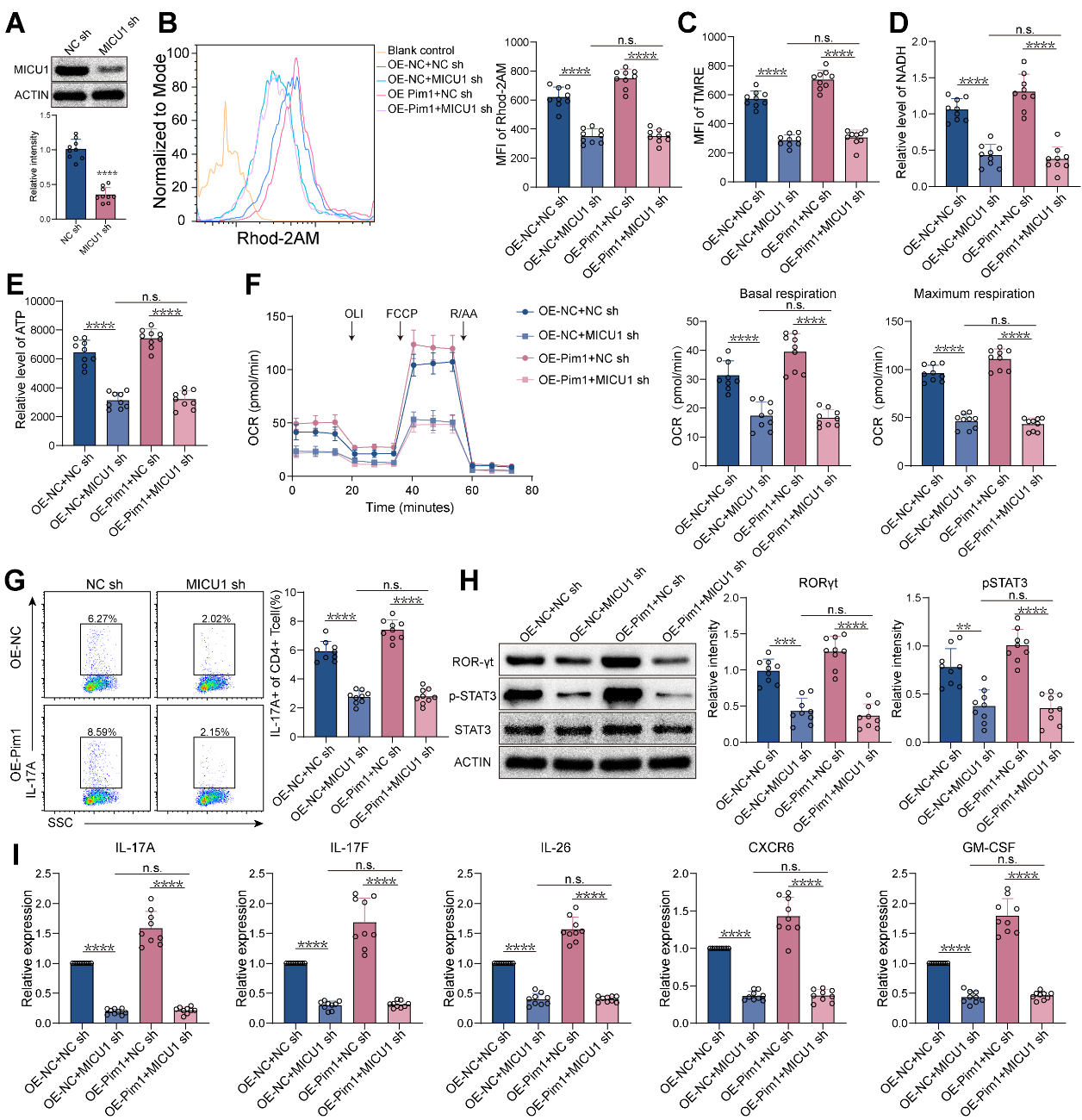
Supplementary Figure 4. Pim1 promotes mito-Ca2+, OXPHOS and Th17 cell differentiation through MICU1**

**A.** The knockdown efficiency of MICU1 in CD4+ T cells (n=9). **B.** MFI of Rhod-2AM in MICU1-knockdown cells in the presence or absence of Pim1 overexpression (n = 9). **C-F.** MFI of TMRE (C) and relative levels of NADH (D) and ATP (E), and OCR (F) of MICU1-knockdown cells in the presence or absence of Pim1 overexpression (n = 9). **G.** Frequency of Th17 cells among CD4+ cells after MICU1 knockdown in the presence or absence of Pim1 overexpression (n = 9). **H.** Relative protein levels of RORγt and pSTAT3 in MICU1-knockdown cells in the presence or absence of Pim1 overexpression (n = 9). **I.** Relative mRNA levels of Th17 cell-associated pathogenic genes in MICU1-knockdown cells in the presence or absence of Pim1 overexpression (n = 9). The statistical analyses were performed as follows: paired t test (A) and One-way ANOVA followed by Bonferroni’s post hoc comparisons test (B-I).


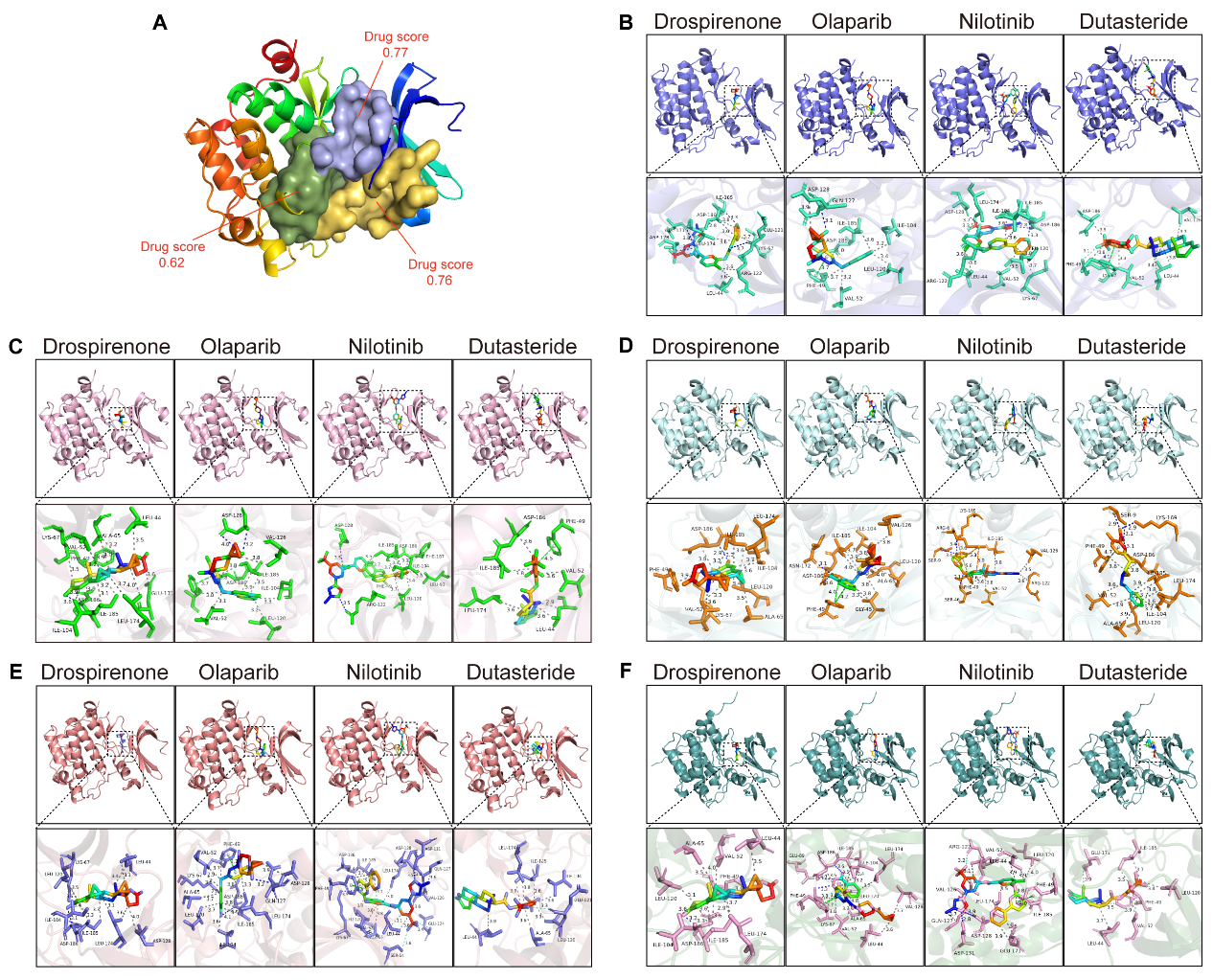


**Supplementary Figure 5. Molecular docking of drugs with different PDB structures of Pim1**

**A.** Drug score analysis of three drug-binding pockets of Pim1 (PDB 1XWS). **B-F.** Molecular docking showing the docking of Drospirenone, Olaparib, Nilotinib and Dutasteride to PDB 2BIK (B), 2BZH (C), 5N4V (D), 6YKD (E) and Pim1AF (F) of Pim1, and the bonds between the four drugs and Pim1. The blue dotted line represents hydrogen bonds, gray represents hydrophobic bonds, green cyan represents halogen bonds and green represents π-stacking.


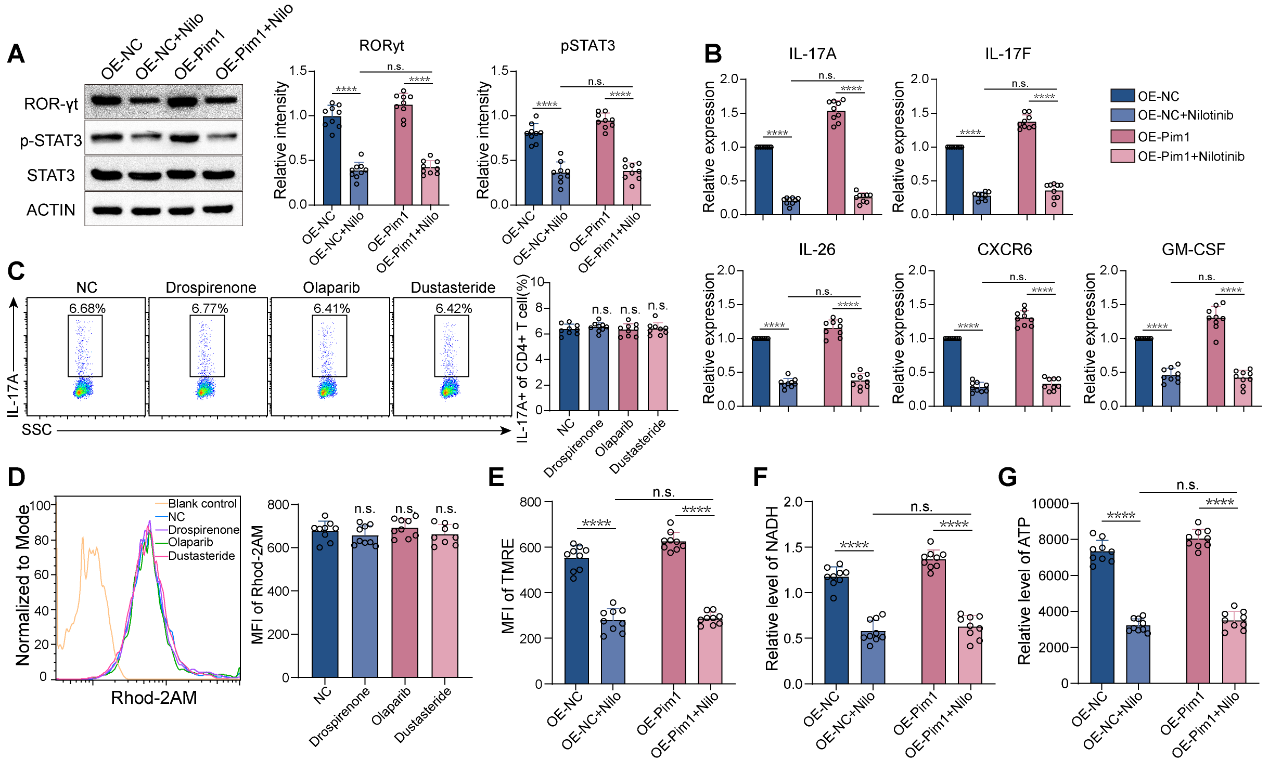


**Supplementary Figure 6. Effects of drugs on Th17 cell differentiation and OXPHOS**

**A.** Relative protein levels of RORγt and pSTAT3 in cells treated with Nilotinib in the presence or absence of Pim1 overexpression (n = 9). **B.** Relative mRNA levels of Th17 cell-associated pathogenic genes in cells treated with Nilotinib in the presence or absence of Pim1 overexpression (n = 9). **C.** Frequency of Th17 cells in CD4+ cells after treatment with Drospirenone, Olaparib, and Dutasteride (n = 9). **D.** MFI of Rhod-2AM of cells treated with Drospirenone, Olaparib and Dutasteride, respectively (n = 9). **E-G.** MFI of TMRE (E) and relative levels of NADH (F) and ATP (G) of cells treated with Nilotinib in the presence or absence of Pim1 overexpression (n = 9). The statistical analyses were performed as follows: One-way ANOVA followed by Bonferroni’s post hoc comparisons test (A-G).


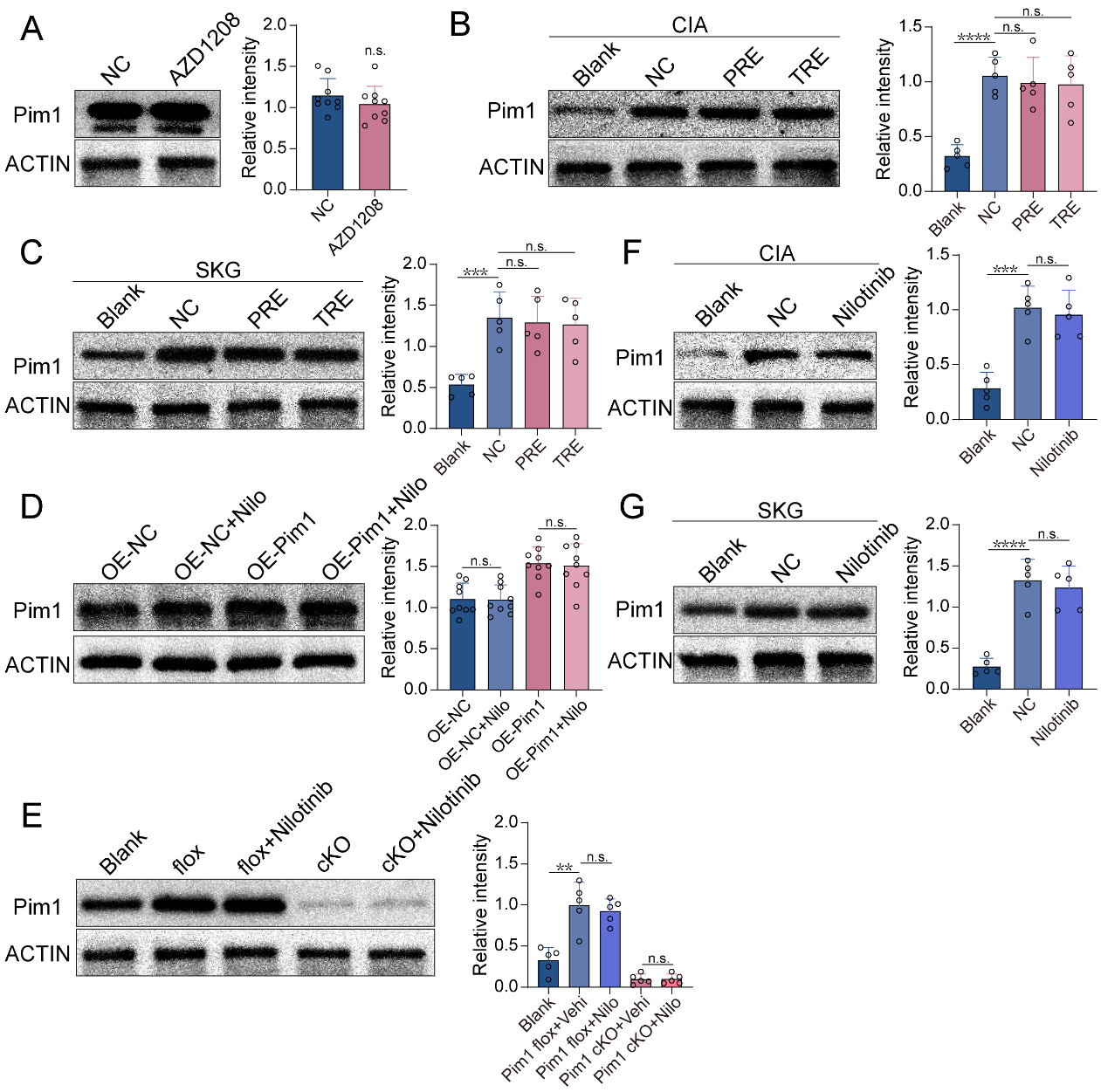
 **Supplementary Figure 7 Effects of AZD1208 and Nilotinib on the expression of Pim1 in CD4+ T cells**

**A.** Pim1 expression in CD4+ T cells treated with AZD1208 in vitro (n=9). **B-C.** Pim1 expression in CD4+ T cells of CIA (B) and SKG (C) mice treated with AZD1208 (n=5). **D.** Pim1 expression in CD4+ T cells treated with Nilotinib in the presence or absence of Pim1 overexpression in vitro (n = 9). **E.** Pim1 expression in CD4+ T cells of Pim1 flox mice and Pim1 cKO mice treated with Nilotinib (n=5). **F-G.** Pim1 expression in CD4+ T cells of CIA (F) and SKG (G) mice treated with Nilotinib (n=5). The statistical analyses were performed as follows: paired t test (A) and One-way ANOVA followed by Bonferroni’s post hoc comparisons test (B-G).


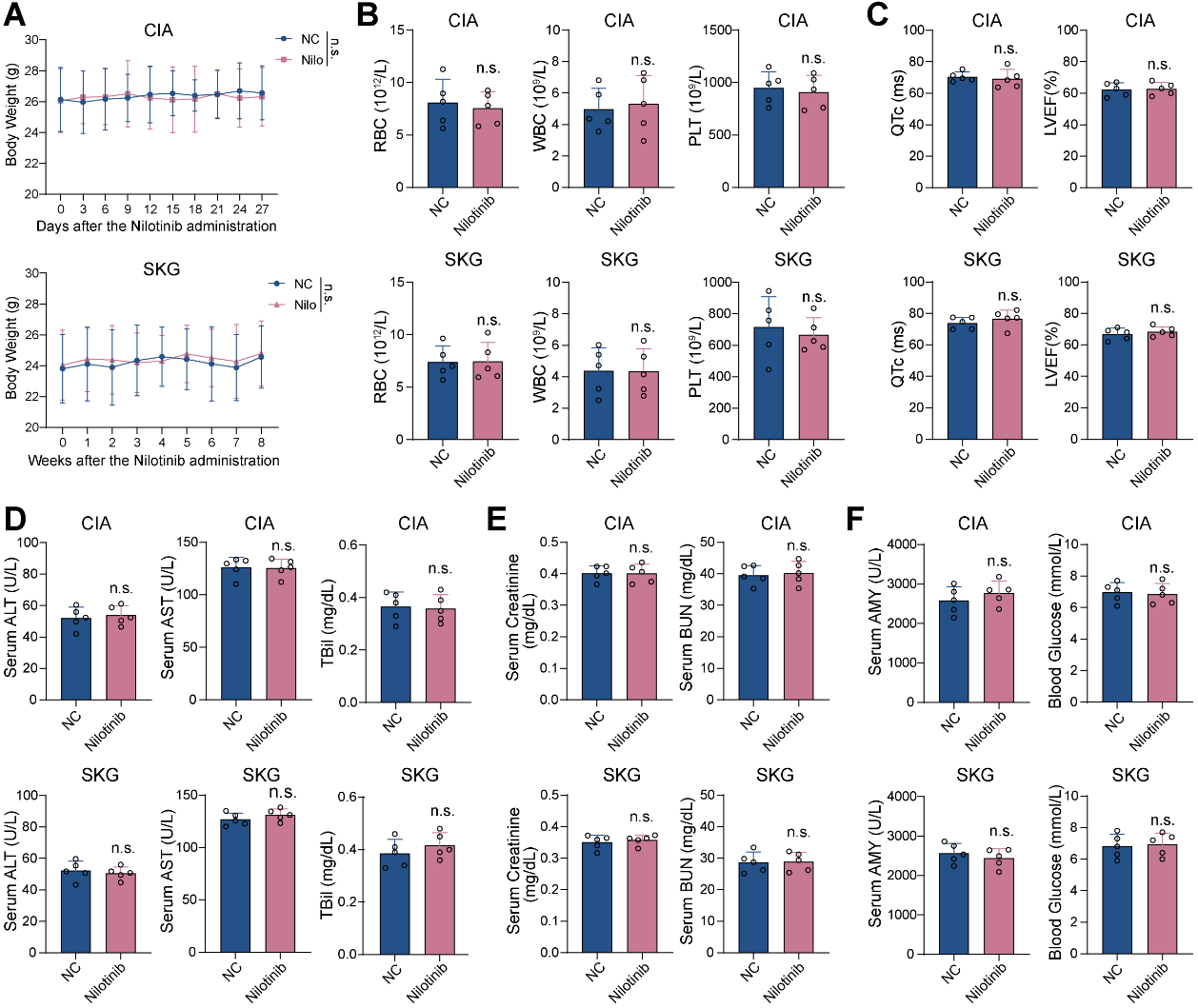
**Supplementary Figure 8. Nilotinib exhibited no significant side effects during inflammatory arthritis treatment**

**A.** Alteration of Body weight after Nilotinib treatment (n=5). **B.** Counts of RBC, WBC and PLT after Nilotinib treatment (n=5). **C.** QTc and LVEF after Nilotinib treatment (n=5). **D.** Activity of serum ALT and AST and concentration of TBil after Nilotinib treatment (n=5). **E.** Concentration of serum creatinine and BUN after Nilotinib treatment (n=5). **F.** Activity of serum AMY and concentration of blood glucose after Nilotinib treatment (n=5). The statistical analyses were performed as follows: Two-way repeated-measures ANOVA (A) and Two-tailed Student’s t-test (B-F).

|  | 1xws | 2bik | 2bzh | 5n4v | 6ykd | Pim1-AF | Mpim1 |
| --- | --- | --- | --- | --- | --- | --- | --- |
| Nilotinib  fda872 | Rank 13；  △G=-10.7 | Rank 20,  △G=-10.9 | Rank 13,  △G=-11.3 | Rank 6,  △G=-11.2 | Rank 8,  △G=-11.2 | Rank 8,  △G=-10.7 | Rank 10,  △G=-9.5 |
| Olaparib  fda350 | Rank 13；  △G=-10.7 | Rank 12,  △G=-11.1 | Rank 2,  △G=-11.9 | Rank 4,  △G=-11.3 | Rank 2,  △G=-11.4 | Rank 4,  △G=-11 | Rank 10,  △G=-9.5 |
| Dutasteride  fda1755 | Rank 4；  △G=-11.3 | Rank 7,  △G=-11.3 | Rank 3,  △G=-11.6 | Rank 26,  △G=-10.7 | Rank 2,  △G=-11.4 | Rank 1,  △G=-11.4 | Rank 57,  △G=-8.8 |
| Drospirenone fda98 | Rank 1；  △G=-12.3 | Rank 1,  △G=-12.5 | Rank 6,  △G=-11.5 | Rank 4,  △G=-11.3 | Rank 11,  △G=-11.1 | Rank 5,  △G=-10.9 | Rank 125,  △G=-8.4 |

**Supplementary Table 1.** **Binding energy and ranking of drugs in dockings with different PDB structures of Pim1**

△G: binding energy (kcal/mol)

**Supplementary Table 2. Clinical characteristics of RA patients and controls**

|  | Synovium samples | |  | PBMCs samples | |
| --- | --- | --- | --- | --- | --- |
|  | **OA** | **RA** |  | **HC** | **RA** |
| Number | 8 | 6 |  | 12 | 12 |
| Female | 5 | 4 |  | 7 | 9 |
| Age (year) | 63.8±5.5 | 61.3±8.0 |  | 60.4±6.3 | 59.5±7.2 |
| CRP (mg/L) | - | 9.7±5.3 |  | - | 7.9±5.8 |
| ESR (mm/h) | - | 27.8±16.6 |  | - | 24.1±16.3 |
| RF positive (%) | - | 83.3 |  | - | 75 |
| Anti-CCP positive (%) | - | 66.7 |  | - | 58.3 |
| Active disease (CDAI≥10, %) | - | 83.3 |  | - | 66.7 |

CRP: C-reactive protein

ESR: erythrocyte sedimentation rate

RF: rheumatoid factor

Anti-CCP: anti-cyclic citrullinated peptide

CDAI: Clinical Disease Activity Index

**Supplementary Table 3. Clinical characteristics of AS patients and controls**

|  | Enthesis samples | |  | PBMCs samples | |
| --- | --- | --- | --- | --- | --- |
|  | **LDH** | **AS** |  | **HC** | **AS** |
| Number | 8 | 4 |  | 12 | 12 |
| Female | 3 | 0 |  | 5 | 4 |
| Age (year) | 54.9±7.3 | 57.8±8.6 |  | 49.3±7.8 | 48.2±10.0 |
| CRP (mg/L) | - | 12.1±7.0 |  | - | 10.3±6.9 |
| ESR (mm/h) | - | 29.8±14.9 |  | - | 27.1±20.9 |
| Active disease (BASDAI≥4, %) | - | 100 |  | - | 58.3 |

BASDAI: Bath Ankylosing Spondylitis Disease Activity Index

**Supplementary Table 4.** **Primer sequences used in this study**

| Gene | Forward primer  (5’-3’) | Reverse primer  (3’-5’) |
| --- | --- | --- |
| Mouse RORγt | GACCCACACCTCACAAATTGA | AGTAGGCCACATTACACTGCT |
| Mouse IL-17A | GGCCCTCAGACTACCTCAAC | TCTCGACCCTGAAAGTGAAGG |
| Mouse T-bet | AGCAAGGACGGCGAATGTT | GTGGACATATAAGCGGTTCCC |
| Mouse GATA3 | CTCGGCCATTCGTACATGGAA | GGATACCTCTGCACCGTAGC |
| Mouse Foxp3 | ACCATTGGTTTACTCGCATGT | TCCACTCGCACAAAGCACTT |
| Mouse Hprt1 | CCTGGCGTCGTGATTAGTGAT | AGACGTTCAGTCCTGTCCATAA |
| Human Pim1 | GAGAAGGACCGGATTTCCGAC | CAGTCCAGGAGCCTAATGACG |
| Human IL-17A | GCTGTCGATATTGGGGCTTG | GGGGACAGAGTTCATGTGGTA |
| Human IL-17F | GCTGTCGATATTGGGGCTTG | GGAAACGCGCTGGTTTTCAT |
| Human IL-26 | GCTGTTAGTCACTCTGTCTCTTG | GGACAATGTTCCCCTTGGGTA |
| Human CXCR6 | GACTATGGGTTCAGCAGTTTCA | GACTATGGGTTCAGCAGTTTCA |
| Human GM-CSF | TCCTGAACCTGAGTAGAGACAC | TGCTGCTTGTAGTGGCTGG |
| Human Hprt1 | CCTGGCGTCGTGATTAGTGAT | AGACGTTCAGTCCTGTCCATAA |
